# Capturing the Resistome: a Targeted Capture Method To Reveal Antibiotic Resistance Determinants in Metagenomes

**DOI:** 10.1128/AAC.01324-19

**Published:** 2019-12-20

**Authors:** Allison K. Guitor, Amogelang R. Raphenya, Jennifer Klunk, Melanie Kuch, Brian Alcock, Michael G. Surette, Andrew G. McArthur, Hendrik N. Poinar, Gerard D. Wright

**Affiliations:** a David Braley Centre for Antibiotic Discovery, McMaster University, Hamilton, Ontario, Canada; b Michael G. DeGroote Institute for Infectious Disease Research, McMaster University, Hamilton, Ontario, Canada; c Department of Biochemistry and Biomedical Sciences, McMaster University, Hamilton, Ontario, Canada; d McMaster Ancient DNA Centre, Department of Anthropology and Biochemistry, McMaster University, Hamilton, Ontario, Canada

**Keywords:** antibiotic resistance, resistome, sequencing, targeted capture

## Abstract

Identification of the nucleotide sequences encoding antibiotic resistance elements and determination of their association with antibiotic resistance are critical to improve surveillance and monitor trends in antibiotic resistance. Current methods to study antibiotic resistance in various environments rely on extensive deep sequencing or laborious culturing of fastidious organisms, both of which are heavily time-consuming operations. An accurate and sensitive method to identify both rare and common resistance elements in complex metagenomic samples is needed. Referencing the sequences in the Comprehensive Antibiotic Resistance Database, we designed a set of 37,826 probes to specifically target over 2,000 nucleotide sequences associated with antibiotic resistance in clinically relevant bacteria. Testing of this probe set on DNA libraries generated from multidrug-resistant bacteria to selectively capture resistance genes reproducibly produced higher numbers of reads on target at a greater length of coverage than shotgun sequencing. We also identified additional resistance gene sequences from human gut microbiome samples that sequencing alone was not able to detect. Our method to capture the resistome enables a sensitive means of gene detection in diverse environments where genes encoding antibiotic resistance represent less than 0.1% of the metagenome.

## Abstract (precis)

Identification of the nucleotide sequences encoding antibiotic resistance elements and determination of their association with antibiotic resistance are critical to improve surveillance and monitor trends in antibiotic resistance. Current methods to study antibiotic resistance in various environments rely on extensive deep sequencing or laborious culturing of fastidious organisms, both of which are heavily time-consuming operations.

## INTRODUCTION

Antimicrobial resistance (AMR) is one of the most pressing challenges of the 21st century that poses a threat to modern medicine and food security (1). The challenge of AMR is amplified by the movement of genes between bacteria, coupled with the movement of people and goods across the planet (2–4). One of the gaps in addressing the antibiotic resistance crisis is a lack of suitable tools to catalog the complete resistome (the entire AMR gene contingent) in various environments and associated microbiomes. Detecting the resistome of an individual bacterium, a microbiome, and other environmental settings (sediment, hospitals, etc.) will aid in tracking the spread of resistance and monitoring the emergence of new resistance alleles associated with the use of antibiotics or other bioactive compounds (5–10). This information can guide antibiotic use, in addition to informing stewardship programs and public health decisions.

Profiling the resistomes of bacteria that are culturable is reasonably straightforward using whole-genome sequencing followed by analysis using algorithms, such as the Resistance Gene Identifier (RGI) in the Comprehensive Antibiotic Resistance Database (CARD) (11). In metagenomes, where resistance determinants are relatively rare, deep sequencing, requiring millions of sequencing reads, followed by careful filtering is needed. This resource-intensive strategy can be alleviated by the targeted detection of selected genes, e.g., via PCR, microarrays, or CRISPR/Cas9-based methods (12–16). However, such highly targeted approaches suffer from the fact that they are rarely comprehensive and generally cannot account for the continual emergence of gene variants and/or completely novel mechanisms of resistance (17–19).

**FIG 1:**
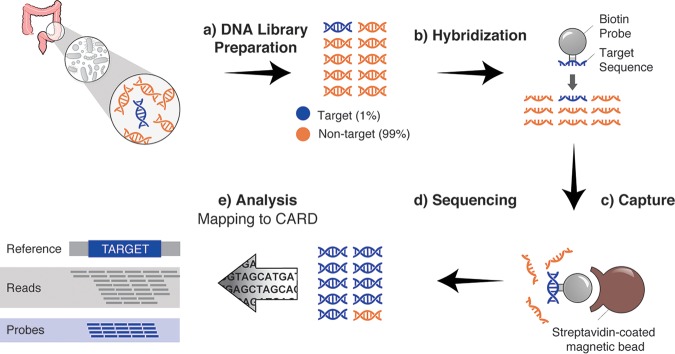
Platform for capture and identification of diverse antibiotic resistance genes. The targeted capture sequencing work flow begins with DNA isolation from a sample of interest (stool from a healthy donor, in this example). (a) DNA is fragmented through sonication and prepared as a sequencing library. (b, c) Target sequences representing less than 1% of the total DNA are captured through hybridization with biotinylated probes and streptavidin-coated magnetic beads. (d, e) The captured and amplified library fragments are sequenced, and reads are analyzed for AMR gene sequence content by mapping to the sequences in CARD.

A more appropriate approach for the identification of resistomes in metagenomes is the use of a probe-and-capture strategy (20). Using this strategy, we and others have captured, sequenced, and reconstructed human mitochondrial sequences as well as the genomes of infectious agents and extinct species from various environments, including highly degraded archeological and historical samples (21–26). In a probe-and-capture experiment, target RNA baits are designed to be complementary to the target DNA sequences of interest. Synthesized probes are biotin labeled and are incubated with the DNA from metagenomic or genomic libraries, where they hybridize to related sequences (Fig. 1a and b). Targets are captured using streptavidin-coated magnetic bead separation, and then the reaction mixtures are pooled and the sequences are determined on a next-generation sequencing (NGS) platform (Fig. 1c to e). This strategy offers significant advantages for the sampling of resistomes in a variety of environments where resistance genes are generally rare and genetically diverse. Indeed, recently, the use of this approach for resistance gene capture has been explored by other groups (27–29). However, these accounts target many other genes that are not rigorously associated with resistance, increasing the sequencing cost and the opportunity for false-positive gene identification.

Here, we chronicle our targeted method for the analysis of antibiotic resistomes. We based our probe set design on stringently curated AMR gene (ARG) sequences from CARD (v1.0.1, 2015), tiled across ARG sequences, combined with rigorous analysis to suppress off-target hybridization. This design enables a more cost-effective and sensitive method to sample the known resistance gene landscape (11). We tested the efficacy of this probe set and our strategy using both a panel of pathogenic bacteria with known resistance genotypes and uncharacterized human metagenomic stool samples. Our method demonstrates the superior design and methodology of the approach, which is readily applicable to both clinical and nonclinical settings.

## RESULTS

### Design and characterization of resistance gene probes.

**FIG 2:**
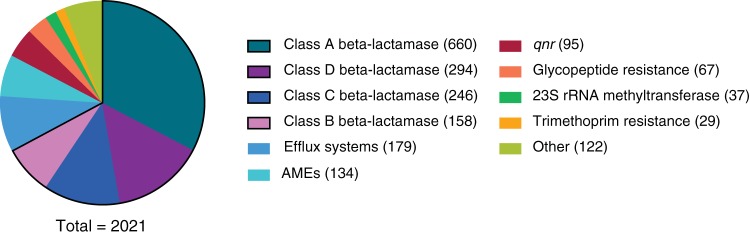
Design of a probe set to target over 2,000 antibiotic resistance genes. Breakdown of resistance gene classes from CARD that are targeted by probes. A legend for the top 10 classes is shown. AME, aminoglycoside-modifying enzymes; *qnr*, quinolone resistance genes. The remaining 122 genes belong to various classes. The beta-lactamase genes make up the majority of genes targeted by probes and are highlighted with a black border.

A set of 80-mer nucleotide probes was custom designed and synthesized through the use of the myBaits platform (Arbor Biosciences, Ann Arbor, MI). The probes (*n* = 37,826) span the protein homolog model of curated ARGs from CARD and represent nucleotide sequences (*n* = 2,021) that are well characterized in the literature. Probes targeting genes for resistance conferred through single point mutations (single nucleotide polymorphisms [SNPs], e.g., sequences contained in the protein variant model in CARD) in chromosomal metabolic genes (including DNA gyrase [*gyrA*] mutations associated with fluoroquinolone resistance and RNA polymerase subunit [*rpoB*] mutations associated with rifampin resistance) were purposefully not included in our design. Of the genes targeted by our probes, 78.03% mirrored the breakdown in CARD, dominated by genes encoding antibiotic inactivation mechanisms and by genes encoding the beta-lactamases (Fig. 2). The majority of the probes (*n* = 24,767) target a single gene, and the remainder target multiple genes ranging up to a maximum of 211 genes (average, 5.96 genes) due to sequence conservation within gene families (see Fig. S1A in the supplemental material). For example, a single probe initially designed to target 80 nucleotides of the beta-lactamase gene *bla*_SHV-52_ also targets an additional 208 genes, including other members of the SHV, LEN, and OKP-A/-B beta-lactamases, due to homology between these nucleotide sequences within AMR gene families. The combination of overlap in the utility of some 80-mer probes and partial hybridization can allow probes to target sequences that are divergent from their reference sequences and thus identify new alleles at the SNP level up to 15% divergence.

At the individual determinant level, the number of probes per gene (average, 105 probes per gene; range, 1 to 309 probes per gene) and the length coverage of a gene (average, 96.20%; range, 3.17% to 100%) vary (Fig. S1B and C). The majority of the targeted genes (2,004/2,021; 99.16%) are covered by at least 10 or more probes (Fig. S1B). Members of the beta-lactamase families (*bla*_CTX-M_, *bla*_TEM_, *bla*_OXA_, *bla*_GES_, *bla*_SHV_) are among the genes with the highest probe coverage. The majority of genes (1,970/2,021) have greater than 80% length coverage by probes, 26 genes have less than 50% length coverage by probes , and only 1 (*mexW*, Antibiotic Resistance Ontology [ARO] ID: 30003031) has less than 5% length coverage by probes (Fig. S1C). Only 28 sequences from CARD have no probe coverage, due to filtering of candidate probes during the design. Overall, this probe set targets ∼1.77 megabases of antibiotic resistance-associated nucleotide sequences and greater than 83% of the nucleotide sequences curated in CARD. Additional metrics of the probe set are given in Fig. S1D to H.

### ARG enrichment from bacterial genomes with a range of antibiotic resistance determinants.

To characterize the sensitivity and the selectivity of this probe set, we conducted a series of control experiments using a panel of sequenced multidrug-resistant Gram-positive and Gram-negative bacteria. The proportion of the genomes targeted by our probe set ranged from 0.21 to 0.97%, consisting of 13 to 65 ARGs representing 102 unique genes among the isolates tested (Table S1). Genomic DNA from four different species was tested individually via enrichment on two different library preparations (the NEBNext Ultra II library preparation versus the modified Meyer and Kircher library preparation) of various insert sizes (average library fragment size range, 396 to 1,257), referred to here as trial 1 and trial 2 (Table S2). Our enrichment approach is insensitive and tractable to different insert sizes, as there was a strong correlation between the read count on targeted regions for bacterial genomes enriched individually between the two trials (Pearson correlation, 0.811 to 0.975) (Table S3; Fig. S2).

**FIG 3:**
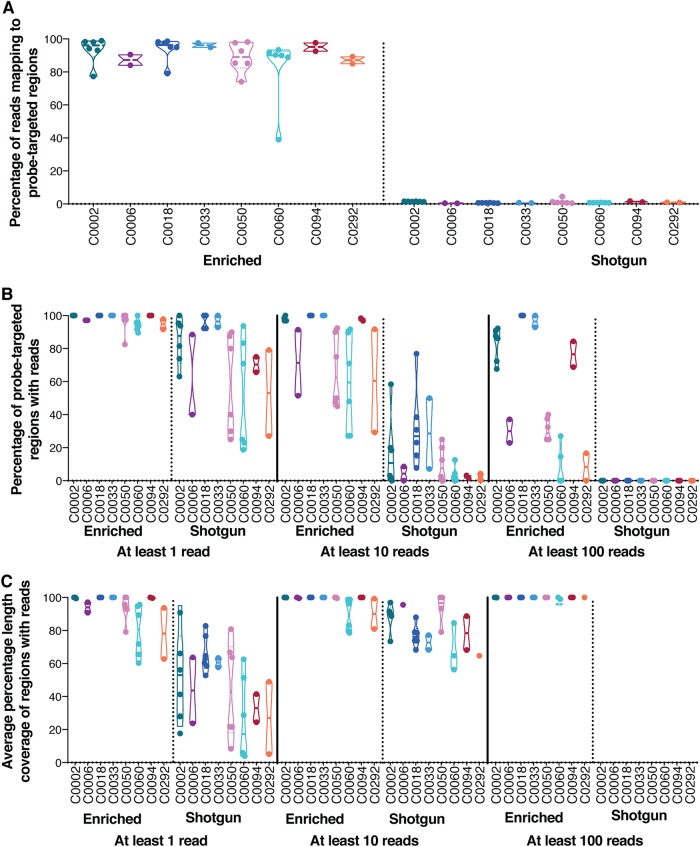
Comparison of enriched and shotgun sequencing results for on-target mapping, recovery, and length coverage. Each point on the graph represents the results of a replicate experiment for either a genome that was enriched individually or a genome pooled with other genomes across both trials. The horizontal line for each isolate represents the mean. (A) Percentage of reads on target for each bacterium tested in various sample types for both enriched and shotgun samples. (B) Percent recovery of regions predicted to be targeted by probes for each bacterial genome tested in both enriched and shotgun samples (1 versus 10 versus 100 reads per probe-targeted region). (C) Average percent length of coverage of probe-targeted regions with reads from isolates tested individually and in pools in both enriched and shotgun samples (1 versus 10 versus 100 reads). If samples did not have any probe-targeted regions with a given read coverage, the results were excluded from panel C. This represents eight samples in the panel labeled “At least 10 reads” (all from the shotgun data [strain C0002, *n* = 1; strain C0050, *n* = 2; strain C0060, *n* = 3; strain C0006, *n* = 1; strain C0292, *n* = 1]), all samples for the shotgun data in the panel labeled “At least 100 reads,” and five samples for the enriched data (strain C0060, *n* = 4; strain C0292, *n* = 1).

This probe set is selective for regions associated with antibiotic resistance in these isolates, given that over 90% of the reads mapped to the respective draft bacterial genomes and the majority (greater than 85% in all cases) of the reads mapped to the small proportion (<1%) of the genome associated with resistance (Fig. 3A; Table S3). We successfully captured 100% of the targeted genes in both library preparation methods with at least 10 reads and with 100% length coverage for the four species of bacteria tested (Table S3). This represents a sensitivity ranging from 0.21% to 0.97% of the total DNA in these samples, with successful enrichment of regions as small as 97 bp (*mexW* in Pseudomonas aeruginosa C0060 with a probe coverage of 2 had greater than 10 reads in both trials) and 80 bp (*crp* in Klebsiella pneumoniae C0050 had greater than 100 reads in both trials). Other genes that had low probe length coverage included *mdtA* (22.4% coverage by 11 probes) in Escherichia coli C0002, which still retained over 100 reads in both trials, and a 140-bp region of *aad(6)* (16.8% coverage by 4 probes) in Staphylococcus aureus C0018 that was recovered with over 1,000 reads in both trials.

### Successful enrichment of ARGs in mock metagenomes.

Genomic DNA from multiple bacteria was pooled at various ratios of 4 or 8 isolates, with the sequences of some bacteria representing less than 10% of the total mock metagenome (Table S4). In 28/32 enrichments, 80% or more of the sequencing reads mapped to probe-targeted regions within the individual bacterial genome regardless of the pooling ratios (Fig. 3A; Table S5). The one exception was trial 1, pool 2 (enriched), where on-target mapping was not as effective (∼70%); nevertheless, even the results of this trial remained over 50-fold better than those of the trial with the shotgun-sequenced samples (shotgun samples) (Table S5). In all shotgun samples, the percentage of reads on target never exceeded 5%, and in 31/32 cases, it was less than 2% of the total sequencing data (Fig. 3A; Table S5).

At the isolate level, the percentage of the mock metagenome that was represented by probe-targeted regions in an individual isolate ranged from 0.0015 to 0.63% of the total DNA (Table S4; Fig. S3). In 21/32 enriched cases, over 90% of the probe-targeted regions were captured by 10 reads or more (Fig. 3B; Table S5). In contrast, none of the shotgun-sequenced samples recovered more than 80% of the probe-targeted regions with at least 10 reads. The cases in which enrichment underperformed were associated with two species in particular: K. pneumoniae and P. aeruginosa (Fig. 3B; Table S5). We defined the sensitivity of detection of AMR for a given isolate to be the percentage of total DNA represented by probe-targeted regions of a given genome at which greater than 90% of the probe-targeted regions were recovered with at least 10 reads. These values ranged from 0.033% for S. aureus C0018 to 0.11% for P. aeruginosa C0060 (Fig. S3). With these bacterial species tested, our probe set could successfully capture the resistome of these isolates, which represents less than 0.1% of the total DNA and even less at the individual gene level.

### Target gene recovery from mock metagenomes by enrichment exceeds that by shotgun sequencing.

**FIG 4:**
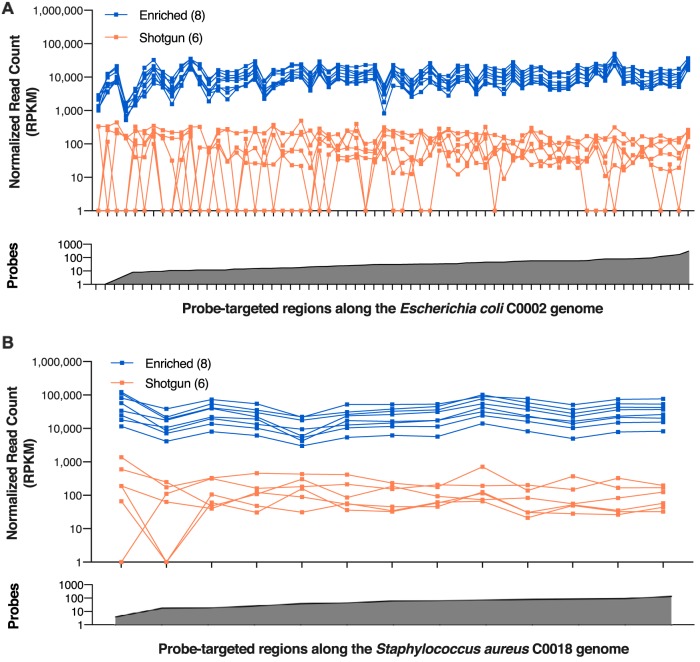
Enrichment results in higher read counts for antibiotic resistance genes than shotgun sequencing. Normalized read counts at each probe-targeted region within the Escherichia coli C0002 genome (A) and Staphylococcus aureus C0018 genome (B) in enriched and shotgun samples, including individual and mock metagenomes of multiple isolates, are shown. Among the enriched and shotgun pairs, reads were subsampled to equal depths and mapped to the individual isolate’s genome. Read counts were normalized by the number of reads mapping per target length (in total number of reads per kilobase per million [RPKM]). The predicted number of probes for each region along the genome is shown at the bottom of each panel. The *y* axes are in the logarithmic scale.

We recovered significantly more targeted genes with at least 1, 10, or 100 reads mapping (mapping quality ≥ 41 MAPQ, length ≥ 40 bp) by enrichment than by shotgun sequencing (Fig. 3B; Table S5). Furthermore, the average percent coverage of the probe-targeted regions with at least 1, 10, or 100 reads in all isolates enriched individually or in pools was always higher than that for the shotgun samples and ranged from being 1.05- to 18.3-fold higher (Fig. 3C; Table S5). For all genomes in all pooled libraries across both trials, the average normalized read count and the depth of the reads on probe-targeted ARGs from enriched libraries were over 50 times (57.09 to 25,683.42) higher than those from the shotgun sequencing control (Table S5). In 31/32 cases, the fold increase in read counts exceeded 2 orders of magnitude and was over 4 orders of magnitude for some probe-targeted regions (Table S5). The one case that did not conform (from trial 1, pool 2; see above) reflects a minor and nonreproducible variability in the quality of the capture for unknown reasons. Nonetheless, there was a clear distinction between the shotgun and enriched samples, with the enriched data showing a more consistent agreement between normalized read counts per probe-targeted region than the shotgun data (Fig. 4). A similar trend was observed when the raw read counts for each sample were used (Fig. S4).

### ARG analysis of a human gut metagenome.

In order to test the efficacy and the reproducibility of our enrichment in more complex samples, we performed enrichments on replicates from metagenomic libraries with DNA isolated from a healthy individual’s stool sample. We compared the results of the experiments with those of traditional shotgun sequencing, whereby selected libraries were sequenced to a depth of over 3.5 million paired reads (Table S2). We included a series of positive-control enrichments with genomic DNA from E. coli C0002, which was previously used for enrichment with the mock metagenomes. In all cases, we identified the same genes, with a consistent number of reads mapping among these replicate enrichments (when subsampled to equal depths among sets), proving reproducibility regardless of the probe and library ratio (Table S6; Fig. S5). Within each set, we found an excellent correlation with the previous results seen with E. coli C0002 (Pearson correlations, >0.923 for all pairs in set 1, >0.924 for all pairs in set 2, and >0.901 for all pairs in set 3) (Fig. S5).

Across the enriched gut microbiome samples with the full number of reads and no filters, on average, 50.69% of reads mapped to sequences from CARD and 68 genes with at least 10 reads were identified, whereas 0.03% of reads mapped, on average, and 32 genes with at least 10 reads were identified in the shotgun libraries (Fig. S6A and B; Table S7). We found significantly more genes with at least 1, 10, and 100 reads from each enriched sample than from the shotgun samples, and the average percent coverage of a gene by the number of reads in the enriched samples was 1.5-fold higher (Fig. S6B and C). When subsampled to the same depth as their enriched pairs (between 22,324 and 149,320 reads), we identified, on average, 1 (range, 0 to 2) antibiotic resistance determinant with at least 10 reads after filtering in the shotgun samples, making comparisons at this level unrealistic (Table S8). Conversely, when subsampled to the depth of the sample with the lowest enriched read coverage (22,324 reads), we identified, on average, 28 ARGs with at least 10 reads in the enriched libraries postfiltering (Table S8).

### High fold enrichment of ARGs from human stool.

We combined the read counts for genes with at least 10 reads that passed the chosen filters within each set to compare the probe and library ratios in subsampled and full-read samples through both enrichment and shotgun sequencing. With the full number of reads, 24/70 (34.28%) of genes detected overlapped across all enriched libraries (*n* = 27), while we identified 16 genes of a total 32 (50.00%) that overlapped across all the shotgun libraries (*n* = 6) (Tables S7 and S9). When subsampled to the lowest enriched read coverage (22,324 reads), there were no genes that overlapped across all 6 shotgun libraries, while 13/47 (27.66%) of the genes overlapped across all 27 enriched libraries (Table S10). Comparing the subsampled enriched libraries (22,324 reads), the majority (31/34) of the genes missing in at least one sample were those with, on average, less than 20 reads across the 27 libraries (Table S10; Fig. S7). The order of genes with higher read counts (i.e., a higher abundance and a higher gene copy number) was consistent among the enriched and shotgun samples, and there was a more significant discrepancy between the two sets of samples for reads associated with lower-abundance genes (Fig. S7 and S8). Thus, enrichment, in the same way as shotgun sequencing, does not in some way bias the prevalence of the rank order of AMR genes in these samples. Finally, both methods resulted in an excellent correlation among technical replicates individually (Pearson correlations, 0.871 for shotgun samples and 0.972 for enriched samples; Fig. S7 and S8).

**FIG 5:**
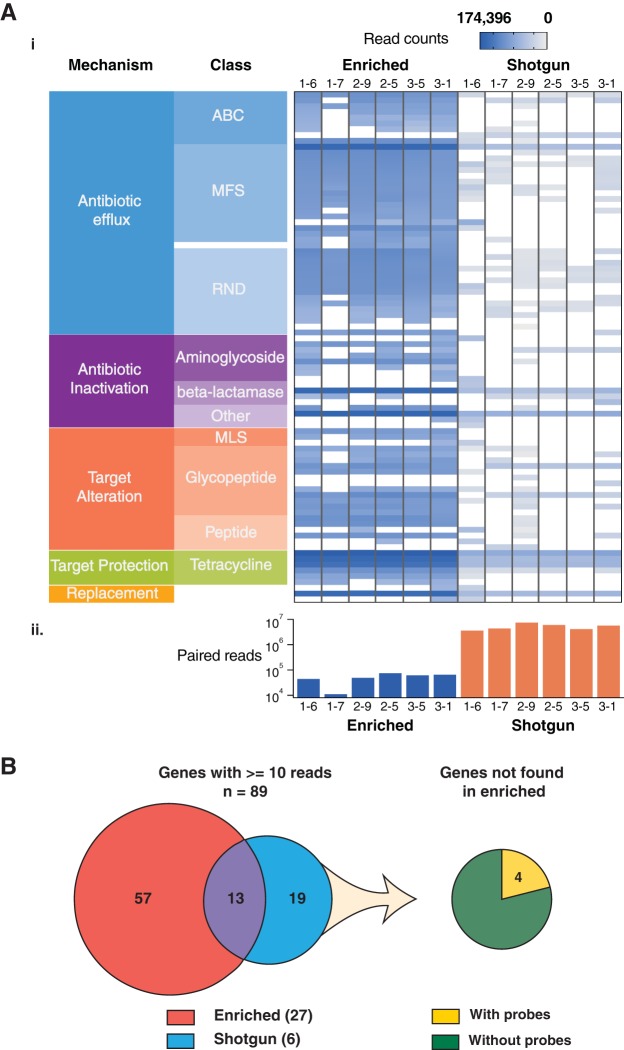
Comparison of resistance elements between enriched and shotgun libraries. For the enriched and shotgun samples, the full number of reads for each sample was mapped to the sequences in CARD using the rgi bwt tool, and the results were filtered for genes with probes mapping with reads with an average mapping quality of ≥11 and a percent length coverage of a gene by reads greater than or equal to 10%. (A) (i) Read counts were normalized per kilobase of reference gene per million reads sequenced (RPKM) and log transformed to produce the heatmap. The rows are grouped based on resistance mechanisms, as annotated in CARD (not all mechanisms and classes are labeled). ABC, ATP-binding cassette antibiotic efflux pump; MFS, major facilitator superfamily antibiotic efflux pump; RND, resistance-nodulation-cell division antibiotic efflux pump; MLS, macrolides, lincosamides, and streptogramins. (ii) The number of reads used for mapping in each sample. (B) (Left) Overlap of genes found with at least 10 reads, a percent coverage greater than or equal to 10%, and an average mapping quality of reads greater than or equal to 11 in the 27 enriched and 6 shotgun samples. Between all samples, enriched or shotgun sequenced, there were 89 genes with reads passing these filters; 13 overlapped, 57 were unique to the enriched samples, and 19 were unique to the shotgun samples. (Right) Of the 19 genes identified only through shotgun sequencing, only 4 of these genes are predicted to be targeted by probes.

We found that the performance of enrichment exceeded that of shotgun sequencing by identifying more unique antibiotic resistance genes at much lower sequencing depths. The enriched samples provided a more diverse representation of ARGs at less than 100,000 paired reads, compared to over 5 million reads in the shotgun samples (Fig. S8 and S9). With the full number of reads in both methods (between 66- and 389-fold more in the shotgun samples than in the enriched samples), the average fold enrichment was >600-fold, and there were still 18 to 50 fewer genes in the shotgun samples than in the enriched samples (Fig. 5A; Table 1). In most cases, there were only a few genes found via shotgun sequencing that were missing in the paired enriched sample (between 9 and 15; 22 unique genes). Only between 1 and 5 of these genes (7 total unique genes) in each sample were predicted to be targeted by probes (Table 1). Of these, only one, *novA*, was missing from all enriched samples but was present in all shotgun samples with >10 reads, a mapping quality of ≥11, and percent length coverage by reads of ≥10%. The other 6 genes (*macB*, *vanRG*, *vanSG*, *smeE*, *cfxA6*, *cepA*) were found in only a few shotgun samples with less than 30 reads and less than 20% read length coverage, on average (Table 1; Table S13). When the two sample types were combined for hierarchical clustering analysis, the enriched libraries clustered separately from the shotgun libraries with a stronger correlation (0.9957 compared to 0.8712 for the shotgun libraries; Fig. S8).

**TABLE 1:** Comparing genes with reads for shotgun and enriched stool library pairs^*a*^

Set	Amt (ng)		Fold difference in no. of reads (enriched vs shotgun)	No. of genes:				Fold enrichment (minimum–maximum)
	Probes	Library		Found in shotgun samples	Found in enriched samples	Overlapping	With probes missing in enriched samples	
1	200	100	389.70	18	49	9	1	1,054.92 (0–10,905.8)
	100	200	82.24	20	25	7	5	1,171.32 (0–6,459.8)
2	400	200	154.93	27	55	12	4	879.87 (0–9,612.1)
	100	100	80.73	23	61	11	1	868.16 (0–8,193.3)
3	100	100	66.67	19	57	9	2	732.16 (0–6,962.7)
	25	50	88.26	22	58	9	2	690.19 (0–7,319.6)

a We mapped the full number of reads from shotgun and enriched pairs to the sequences in CARD using the rgi bwt tool. The results for the samples were filtered for genes with at least 10 reads, those to which probes mapped (only for the enriched samples), an average read mapping quality of ≥11, and an average read length coverage of ≥10%. Filtered genes and their normalized read counts (RPKM) from each enriched sample/shotgun sample pair were combined to compare and determine the fold enrichment.

We then compared the overlap between all 27 enriched samples and the 6 shotgun-sequenced samples and included genes found through shotgun sequencing without any probes mapping. We found a total of 89 genes with at least 10 reads between all libraries, of which 13 overlapped between methods, 57 were unique to the enriched libraries, and 19 were unique to the shotgun libraries (Fig. 5B; Table S13). Of the 19 genes not found in any enriched library, only 4 were predicted to be targeted by probes, while the remaining were not in CARD when the probes were initially designed (*n* = 8 gene sequences) or had probes that were removed during design and filtering (*n* = 7 gene sequences). Of the four genes with predicted probes, *cfxA6* was present in all enriched samples but was filtered out by mapping quality, *vanSG* was present in only 2/6 shotgun samples at less than 20% gene length coverage by reads, and *cepA* was found in enriched samples but at less than 10 reads; finally, we identified *novA* in all shotgun samples but in only a few enriched samples at less than 10 reads and less than 10% read length coverage. Despite the few (*n* = 4) genes that were missing from the enriched samples, even a 200-fold greater sequencing depth of our shotgun libraries could not provide results that match those shown by our enrichment data (Fig. S9).

### Negative-control results.

To track and measure the contamination in the laboratory environment at McMaster University within the Michael G. DeGroote Institute for Infectious Disease Research, commercial kits, environment, and reagents, we included negative controls consisting of a blank DNA extraction and negative reagent controls in enrichments that we processed in a manner identical to that used for our samples in phases 1 and 2. For phase 1 in both trial 1 and trial 2, we found a negligible amount of library DNA in the blank after enrichment, and very few of the sequenced reads were associated with the indexes used for the blank library (between 2.46% and 8.96% of sequenced reads; Tables S2 and S11). Only the blank samples from phase 1, trial 1, and phase 2, set 2, resulted in genes with at least 10 reads mapping (10 and 19, respectively; Table S12).

## DISCUSSION

Increased interest in targeted capture approaches has resulted in the design of probe sets for the detection of viruses, bacteria, and, more recently, antibiotic resistance elements (26–29). Although our study is not the first to employ targeted capture for antibiotic resistance genes, we have focused on a rigorous probe design that includes choosing an appropriate reference database, robust probe set validation, and experimental considerations for enrichment, including reduced input library and probe concentrations (25, 30–33). Our probe design and the application of in-solution targeted capture ultimately result in a cost-effective alternative to shotgun sequencing for identifying antibiotic resistance genes in complex environmental and clinical metagenomes.

### Reference database for probe design and analysis.

CARD was chosen as the reference database for our probe design (v1.0.1) and analysis (v3.0.0) due to its rigorous curation of antibiotic resistance determinants. We excluded some genes (e.g., *gyrA*, EF-Tu genes, etc.) that are likely to be found as homologs across many families of bacteria and that would likely have overwhelmed the probe set and sequencing effort with abundant, nonmutant antibiotic-susceptible alleles. Instead, we chose CARD’s protein homolog model (v1.0.1, *n* = 2,010 genes) to focus our approach on genes that are likely to be acquired (i.e., genes associated with mobile genetic elements) and those that are unique to individual families of bacteria. Therefore, although we were unable to detect resistance conferred by SNPs in chromosomal metabolic genes, our probe set was capable of capturing the vast majority of resistance elements and those that were at a higher risk of being mobile. In future probe designs, the protein variant model of CARD (v1.0.1, *n* = 77 genes; v3.0.0, *n* = 141 genes) can be targeted using probes specific to the regions of a gene associated with a given set of SNPs, but they will need to be carefully tested *in silico* to ensure that they do not enrich unintended targets. Given that in certain populations (e.g., metagenomes) these variant sequences may be less abundant than their susceptible counterparts, careful and rigorous analysis will need to be implemented to identify the relevant variants (i.e., RGI developments).

To address our probe set’s compatibility with a frequently updated database, we chose a more recent version of CARD (v3.0.0, *n* = 2,238 genes) for comparative analysis with our bait set designed in 2015. Since the design of our probes against v1.0.1 of CARD, the database has been updated and includes 264 additional genes. Despite these changes, our probe set targeted the majority (2,021/2,238) of known antibiotic resistance gene sequences from CARD (v3.0.0). In reality, the probes should target sequences with up to 15% nucleotide sequence divergence from a reference sequence, suggesting a wider applicability and target capacity toward newly characterized members of AMR gene families, which often differ from other members by only a few nucleotides. Of the 264 genes added to CARD (v3.0.0), our existing probes capture 75 of these genes that are sufficiently similar to other targeted members of the same AMR gene family (e.g., genes for aminoglycoside acetyltransferases, chloramphenicol acetyltransferases, and beta-lactamases [*bla*_ACT,_ *bla*_CARB,_ *bla*_CMY,_ *bla*_LEN,_ *bla*_NDM,_ *bla*_OXA,_ *bla*_PDC._ *bla*_SHV,_ *bla*_TEM,_ *bla*_VEB_]). Of the remaining genes, the sequences of 60 have been newly identified since 2015, and the other genes, although mentioned in the literature prior to 2015, were added due to increased efforts of curation of CARD.

Other approaches targeting ARGs have included probes for species identifiers, plasmid markers, and biocide or metal resistance (27–29). These probe sets range in target capacity from 5,557 genes (3.34 Mb) (28) to over 78,600 genes (88.13 Mb) (27) and comprise up to 4 million probes (29). Other strategies involve designing one probe per gene, tiling probes across a gene without overlap (1× coverage), or interprobe distances of up to 121 nucleotides (28, 29). Our approach is more conservative in probe design (1.77 Mb for 2,021 genes), but the dense tiling allows for more probes per gene (99.16% of genes had greater than 10 probes) and an increased depth of probe coverage (average, 9.47 times). We believe that the design approach increases the specificity, sensitivity, and likelihood of capturing rare DNA molecules common in complex metagenomes (34). We also performed extensive filtering of candidate probes against the human genome and other eukaryote, archaeal, and weakly matching bacterial sequences to provide a probe set that is bacterial ARG specific and avoids off-target hybridization. Focusing on one highly curated database of antibiotic resistance determinants (CARD) increases the likelihood of capturing bona fide sequences that are associated with known resistance and reduces the overall cost of the probe set and sequencing effort. When updates to CARD are released or if additional markers are of interest, probes can easily be designed and added to the existing probe set.

### Experimental considerations in targeted capture methods.

For our trials, we tested amounts of inputs (25 ng to 400 ng) significantly smaller than the amount recommended by the manufacturer (up to 2 μg of DNA for metagenomic samples), setting our method apart from other methods for the targeted capture of AMR genes (27, 28). Others have looked at reducing the amount of input DNA from the manufacturer’s recommended amount of 3,000 ng to 500 ng and saw no significant differences in results (35). Despite a 16-fold reduction in the DNA input amount (25 ng versus the recommended 2,000 ng), we saw no visible differences in the order of genes captured in the stool sample, and the normalized read counts were comparable among the different library and probe amounts, suggesting that our approach is robust to tremendous fluctuations yet still identifies all antibiotic resistance genes in samples with a low DNA yield (e.g., clinical and environmental samples). Furthermore, a lower input concentration of probes also reduces the cost per reaction.

### Reproducibility, sensitivity, and performance with clinical isolates.

The sensitivity of our probe set was tested using individual bacterial genomes and mock metagenomes, wherein the percentage of total DNA represented by probe-targeted antibiotic resistance genes ranged from 0.0015% to 0.97%. A successful enrichment in our trials was considered when greater than or equal to 90% of the probe-targeted regions with 10 or more reads were captured. When tested individually, enrichment was able to successfully capture all probe-targeted ARGs (100% with more than 10 reads) in the four bacterial species tested, with >85% of sequenced reads mapping to the targeted regions (<1%) of the genome. With the mock metagenomes, the probe-targeted regions of each isolate represented a smaller proportion, and there were 11 cases in which enrichment was not successful under the above-described criterion. In 7 instances, the given isolate represented less than 10% of the total pool, and many of the probe-targeted regions that were missing were short (<200 bp), and less than 5 probes for these regions were designed (see Table S4 in the supplemental material). One particular predicted resistance gene that was not captured in 2 cases, *fosA2* (ARO:3002804) in K. pneumoniae C0050, retained good probe coverage, despite a low percent identity (71.32%) to the CARD reference sequence. The poor performance in enrichment may suggest the limit of sequence similarity (>30%) that can be captured by probes designed against a single reference sequence. In addition, the high GC content of certain genes in the K. pneumoniae isolates and of many regions of the P. aeruginosa isolates (average GC content, 67%) likely reduced the capture efficiency in the more complex pooled samples, resulting in less than 10 reads for targeted genes. The conditions of hybridization may need to be further optimized for targets with higher GC contents. Regardless of this limitation, the enriched data provide significantly more read coverage for antibiotic resistance genes at a lower depth of sequencing than shotgun sequencing of these mock metagenomes.

### Standardization and controls in metagenomics.

Standardization (including reproducibility) in enrichment studies remains sorely lacking. In this study, we attempted to reduce bias and assess enrichment by using the same DNA extract, library preparations, and enrichment in triplicate. Even among replicate libraries and shotgun sequencing runs, the differences in the number of genes identified at various sequencing depths highlight the inherent variability in metagenomics (Fig. S8). The positive control (E. coli C0002), processed alongside the other samples, ensured that our methodology and probes were performing optimally at the time of hybridization. We also introduced negative controls to measure the extent of exogenous DNA contamination, which is ubiquitous in all laboratory settings and reagents (36, 37). Between 86.07 and 100% of the sequenced reads from our negative controls had corresponding index sequences from experimental samples, suggesting that DNA exchange among samples during enrichment or cross-contamination is the primary concern with our method (Tables S2 and S11). Notably, the genes identified in the results for the blanks not arising from cross-contamination and also found in the enriched and shotgun results are commonly associated with bacteria identified in negative controls in microbiome studies (mainly Escherichia coli) and encode efflux systems or other intrinsic resistance determinants (*mdtEFHOP*, *emrKY*, *cpxA*, *acrDEFS*, *pmrF*, *eptA*, *tolC*). The two genes that were unique to the results for the blanks [*drfA17* had 11 reads with 85.86% coverage; *aph(3ʺ)-Ib* had 16 reads with 57.46% coverage] are associated with mobile genetic elements in *Enterobacteriaceae*, and the latter has previously been associated with laboratory reagent contamination (38, 39). Despite the use of standard methods to control for contamination (i.e., filter pipette tips, PCR cabinets, and sterile DNA- and RNA-free consumables), we still found limited contamination, likely stemming from reagents and/or the surrounding laboratory environment, further highlighting the importance of negative controls in all targeted capture experiments and meticulous reporting and publishing of a laboratory-based resistome (Table S6) (36, 37, 40).

### Enrichment in the gut microbiome.

Our enrichment of resistance genes in the human gut microbiome samples resulted in a higher average percentage on target (50.69%) compared to that obtained by other published capture-based methods, 30.26% (range, 20.27% to 41.83%) (27) and a median of 15.8% (range, 0.28% to 68.2%) (28), highlighting the increased specificity of our probe design. Overall, our probe set and method identified a greater diversity of antibiotic resistance genes in the human gut microbiome, despite having been sequenced at a 66- to 389-fold lower depth than the shotgun-sequenced correlate. With a reduced depth of sequencing, it is evident that enrichment offers more valuable information in terms of both the number of genes with reads as well as the depth and breadth of coverage of those genes (Fig. 5).

Although shotgun sequencing can provide additional information on other functions and genes of interest, our targeted capture provides a more robust and a more reproducible profile of antibiotic resistance genes from metagenomes at a fraction of the sequencing cost. Only a few genes were absent in the enriched libraries but present in the shotgun libraries. In the case of *novA*, which has a 70.51% GC content, there was a gap in the tiling of probes across the gene, and the hybridization conditions were perhaps not sufficient to capture this gene by our method. Additional probes or denser tiling along high-GC-content (>65%) sequences may facilitate successful capture. Another gene that we could not identify, the variant of the *vanS* (GC content, 36.7%) sensor from vancomycin resistance gene clusters, was covered by less than 20 reads in the shotgun samples, suggesting a very low abundance in the metagenome. Finally, the beta-lactamase genes *cepA* and *cfxA6* had been excluded from the enriched results after filtering due to low mapping quality or less than 10 reads. The low mapping quality suggests that reads are mapping to other beta-lactamase genes in the reference database.

All current methods to detect antibiotic resistance genes have limitations. Culturing, although time-consuming, remains the standard for diagnosing infections through the identification of both the pathogen and its susceptibility to a panel of antibiotics. Other biochemical techniques have been developed but are often organism specific and require additional assays for confirming ARGs (41). When studying the microbiome and the resistome of various environments, a culture-based approach is not feasible and, thus, high-throughput methods are needed (19). Sequencing-based approaches (i.e., PCR-based assays, microarray-based assays, and in-solution targeted capture) and quantitative PCR (qPCR) methods offer selective and sensitive means to identify a larger contingent of antibiotic resistance genes than other methods but can be (or are designed to be) heavily biased or selective. While PCR is highly sensitive, many panels for AMR genes target only a range of between 200 and 400 genes (42). As we have shown here, probe-based hybridization methods enable the detection of over 2,000 ARGs in a single assay.

Compared to the other probe sets designed for AMR (27–29), ours offers a highly curated specific set of probes with a high coverage of ARGs and works exceptionally well on samples with low inputs. We have also included crucial controls to validate our findings. Whereas shotgun sequencing requires millions of reads to detect a few antibiotic resistance genes, we have shown that targeted capture can detect the same genes and more with ∼50-fold less sequencing effort. A reduced amount of sequencing allows more samples to be processed per individual sequencing run, reducing sequencing costs overall and increasing throughput. One limitation to targeted approaches is that the probe design relies on known reference sequences, while shotgun sequencing can reveal additional information not captured by the probes, but at an added cost (depth). All sequencing-based methods are limited in the inability to characterize completely novel antibiotic resistance determinants, whereas a functional metagenomics approach is ideal in this regard (19).

In conclusion, we have rigorously measured the performance of our probe design and methods to satisfy many of the parameters in targeted capture routinely discussed (43). The sensitivity and specificity of our probe set are evident from the consistently high percentage of reads on target and the high recovery of probe-targeted sequences representing <0.1% of the total DNA. Our approach results in the uniform recovery of ARGs across bacterial genomes and is reproducible between library preparations. We believe that our targeted capture serves a critical role in the surveillance and detection of ARGs across complex environmental settings, hospitals, and clinics. Profiling of these resistomes will provide invaluable information that can be used to target antibiotic and resistance inhibitor discovery but that at the same time can be used to keep abreast of the rapidly shifting rise of local and global antibiotic resistance.

## MATERIALS AND METHODS

### Nucleotide probe design and filtering to prevent off-target hybridization.

Our reference for probe design was the protein homolog model of antibiotic resistance determinants (*n* = 2,129) from CARD (v1.0.1, released 14 December 2015) (11). Using PanArray (v1.0) software, we designed probes with a length of 80 nucleotides across all genes with a sliding window of 20 nucleotides and acceptance of 1 mismatch across probes (32). To prevent off-target hybridization between the probes and nonbacterial sequences, the candidate set of probe sequences (*n* = 38,980) was compared against the human reference genome and GenBank’s nonredundant nucleotide database through BLAST (blastn) analysis (44, 45). Probes with high sequence similarity (>80%) and probes with high-scoring segment pairs (HSPs) of greater than 50 nucleotides of a possible 80 were discarded (human genome sequences, *n* = 158; eukaryotic sequences, *n* = 1,617; viral sequences, *n* = 774; archaeal sequences, *n* = 30). Probes with HSPs of less than 50/80 nucleotides to bacterial sequences were additionally discarded, resulting in a set of 32,066 probes. The candidate list was further filtered to omit probes that had bacterial HSPs that had <95% identity, resulting in a candidate list of 21,911 probes.

### Optimizing probe density and redundancy.

Probe sequences, along with 1 to 100 nucleotides upstream and downstream of the probe location on the target gene, were sent to Arbor Biosciences (Ann Arbor, MI) for probe design. These sequences are contained within the open reading frame of the target gene and allow probe sequences to be modified, if needed (i.e., polynucleotides at the termini), ensuring that the desired probe coverage of the target genes is attained. An additional 80 nucleotide probes were created across the candidate probe and flanking sequences at a tiling density of four times, resulting in 226,440 probes. Sequences with 99% identity over 87.5% of their length were collapsed using the USEARCH program (settings, usearch -cluster_fast -query_cov 0.875 -target_cov 0.875 -id 0.99 -centroids), resulting in a set of 37,826 final probes (46). Filtering against the human genome was performed by a method similar to that described above; no probes were found to be similar. Arbor Biosciences (Ann Arbor, MI) synthesized this final set of 37,826 80-nucleotide biotinylated single-stranded RNA probes by use of the custom myBaits kit (catalog number 300248; Arbor Biosciences, Ann Arbor, MI).

### Probe assessment and predicted target genes.

To predict the genes that can be targeted by the probes, a Bowtie2 program (the settings used included bowtie2 --end-to-end -N 1 ‘-L 32’ -a) (47) alignment was performed to compare the set of 37,826 probe sequences to the 2,238 nucleotide reference sequences of the protein homolog models in CARD (v3.0.0, released 11 October 2018). The alignment file was manipulated through the use of samtools and bedtools to determine the number of instances that a probe mapped to a nucleotide sequence in CARD, the fraction of each gene sequence covered by probes (length coverage by probes), and the depth of coverage by probes of each gene (bedtools genomecov, bedtools coverage -mean) (48, 49). The GC content of the probe sequences and the nucleotide sequences in CARD was calculated using a Python3 script from https://gist.github.com/wdecoster/8204dba7e504725e5bb249ca77bb2788. The melting temperature (*T_m_*) was determined using the OligoArray function melt.pl (settings, -n RNA, -t 65 -C 1.89e^−9^) (50). We used Prism (v8) software for macOS (GraphPad Software) to generate the plots shown in Fig. S1 in the supplemental material.

### Bacterial isolates, samples, and DNA extraction.

Clinical bacterial isolates were obtained from the IIDR clinical isolate collection, which consists of isolates from the core clinical laboratory at the Hamilton Health Sciences Centre (Table S1). Genomic DNA was isolated from a cell pellet using the PureLink genomic DNA mini kit (catalog number K182002; Invitrogen, Carlsbad, CA). If DNA was not isolated on the same day, we stored the cell pellets at −80°C. While genomic DNA from all other isolates was extracted only once, DNA from a cell pellet of Pseudomonas aeruginosa C0060 was additionally extracted using a varied genomic lysis/binding buffer (30 mM EDTA, 30 mM Tris-HCl, 800 mM guanidine thiocyanate, 5% Triton X-100, 5% Tween 20, pH 8.0). We obtained a human stool sample from a healthy volunteer for the purpose of culturing the microbiome with consent from the Hamilton Integrated Research Ethics Board (HiREB approval number 5513-T). DNA was extracted on the same day following a modified protocol described elsewhere (51). Briefly, samples were bead beaten and centrifuged, and the supernatant was further processed using a MagMax Express 96-well deep well magnetic particle processor from Applied Biosystems (Foster City, CA) with a multisample kit (catalog number 4413022; Life Technologies). DNA was stored at −20°C until it was used for library preparation.

### Isolate genome sequencing.

Library preparation for genome sequencing of the clinical bacterial genomes was completed by the McMaster Genomics Facility in the Farncombe Institute at McMaster University (Hamilton, ON, Canada) using the Nextera XT DNA library preparation kit (catalog number FC-131-1024; Illumina, San Diego, CA). Libraries were sequenced using an Illumina HiSeq 1500 or Illumina MiSeq v3 platform and v2 (2 × 250-bp) chemistry. Paired sequencing reads were processed through a Trimmomatic (v0.39) trimmer to remove adapters, checked for quality using the FASTQC program (http://www.bioinformatics.babraham.ac.uk/projects/fastqc/), and *de novo* assembled using SPAdes (v3.9.0) software (52, 53). The Livermore metagenomics analysis toolkit (LMAT; v1.2.6) was used to identify the bacterial species and screen for contamination or a mixed culture, while the Resistance Gene Identifier (RGI; v4.2.2) from CARD was used on the contigs obtained with SPAdes software to identify perfect (100% match) and strict (<100% match but within CARD similarity cutoffs) hits to CARD’s curated antibiotic resistance genes (54).

### Trials for enrichment.

We performed two phases of experiments. The first was with genomic DNA from cultured multidrug-resistant bacteria (phase 1), and the second was with metagenomic DNA from a human stool sample (phase 2). The two trials in phase 1 differed in their library preparation methods, as described below (the major difference being the library fragment size obtained by sonication). In both trials, we tested genomic DNA from isolates individually (Escherichia coli C0002, Pseudomonas aeruginosa C0060, Klebsiella pneumoniae C0050, and Staphylococcus aureus C0018) (Tables S1 and S3). In addition, various nanogram amounts (based on the absorbance; Thermo Fisher Nanodrop spectrophotometer [Waltham, MA]) of each genome were combined prior to library preparation to create mock metagenomes, referred to as pool 1 (with the genomes of strains C0002, C0018, C0050, and C0060), pool 2 (with the genomes of strains C0002, C0018, C0050, and C0060), and pool 3 (with the genomes of strains C0002, C0018, C0050, C0060, Klebsiella pneumoniae C0006, Staphylococcus aureus C0033, Escherichia coli C0094, and Pseudomonas aeruginosa C0292). The amounts of the genome of each isolate in each pool varied between trials (Table S4). Phase 2 consisted of 3 replicates, referred to as set 1, set 2, and set 3, wherein a DNA extract from one individual human stool sample was split evenly into each set. From these aliquots, we generated 9 individually indexed sequencing libraries and performed capture with various library and probe ratios (Table S3). In all trials and sets, a blank DNA extract was carried throughout library preparation and enrichment, while an additional negative reagent control was introduced during enrichment.

### Library preparation for enrichment sequencing.

Library preparations were performed in a PCR clean hood, using bleached equipment, and the equipment was UV irradiated before use to prevent nonendogenous DNA contamination. Trial 1 library preparations were performed through the McMaster Genomics Facility using the NEBNext Ultra II DNA library preparation kits for Illumina (catalog number E7645L; New England BioLabs, Ipswich, MA). Based on absorbance and fluorometer values (QuantiFluor; Promega, Madison, WI), we sonicated approximately 1 μg of individual bacterial genomic DNA or pools of genomic DNA to 600 bp and prepared dual-index libraries with a size selection for 500- to 600-bp inserts. Postlibrary quality and quantity verification was performed using a high-sensitivity DNA kit for the Agilent 2100 bioanalyzer (catalog number 5067-4626; Agilent Technologies, Santa Clara, CA) and quantitative PCR using a Kapa SYBR Fast qPCR master mix for Bio-Rad machines (catalog number SFBRKB; Sigma-Aldrich, St. Louis, MO), primers for the distal ends of Illumina adapters, and the following cycling conditions: (i) 95°C for 3 min, (ii) 95°C for 10 s, (iii) 60°C for 30 s, (iv) a repeat of steps (ii) and (iii) for 30 cycles total, (v) 60°C for 5 min, and (vi) hold at 8°C. We used Illumina’s PhiX control library (catalog number FC-110-3001; Illumina, San Diego, CA) as a standard for quantification.

In trial 2, the same genomic DNA, except for that of P. aeruginosa C0060, which was reisolated, was used for library construction through a modified protocol (see the supplemental material) (55). Briefly, we performed blunt-end repair, adapter ligation, library size selection, and indexing PCR on ∼200 ng of sonicated DNA (250 to 300 bp). The McMaster Genomics Facility performed library quality control as described above.

### Library preparation from a human stool sample.

We divided one DNA extract from a donor stool sample into three 50-μl aliquots of approximately 3,150 ng each (based on QuantiFluor fluorometer results). DNA was sonicated to 600 bp and split into 9 individual library reaction mixtures (350 ng in 5.55 μl). We prepared dual-index libraries (NEBNext Ultra II DNA library preparation kits for Illumina [catalog number E7645L; New England BioLabs, Ipswich, MA]) with a size selection for 700- to 800-bp library fragments and 6 (set 1), 7 (set 2), or 8 (set 3) cycles of amplification. The McMaster Genomics Facility performed library quality control (with an Agilent 2100 bioanalyzer and by quantitative PCR, as described above). We generated positive-control libraries using Escherichia coli C0002 genomic DNA (40 ng of sonicated DNA) and a negative control with a blank DNA extract.

### Targeted capture of bacterial isolates.

We performed enrichments in a PCR clean hood, with a water bath, thermal cyclers, and heat blocks being located nearby. The probe set was provided by Arbor Biosciences (Ann Arbor, MI) and diluted with deionized water. For enrichment of bacterial genomes in trial 1, we used 100 ng of probes and 100 ng of each library, following the instructions in the myBaits manual (v3; Arbor Biosciences, Ann Arbor, MI), at a hybridization temperature of 65°C for 16 h (see the methods in the supplemental material for more details). After hybridization and capture with Dynabeads MyOne streptavidin C1 beads (catalog number 65001; Thermo Fisher, Waltham, MA), the resulting enriched library was amplified through 30 cycles of PCR (cycling conditions are described in the supplemental material) using Kapa HiFi HotStart polymerase with library-nonspecific primers (Kapa library amplification primer mix [10×]; catalog number KK2620; Roche Canada). A 2-μl aliquot of this library was amplified in an additional PCR for 3 cycles (under the same conditions described above) and then purified. We performed the capture in trial 2 in the same manner described above for trial 1 but applied 17 cycles of amplification postcapture (see the PCR conditions in the supplemental material). The McMaster Genomics Facility performed library quality control as described above. The libraries were pooled in equimolar amounts and sequenced to an average of 94,117 clusters by use of an Illumina MiSeq sequencer (v2; 2 × 250-bp reads). Preenrichment libraries for the mock metagenomes were sequenced in a separate Illumina MiSeq (v2; 2 × 250-bp reads) run from the enriched libraries to an average of 93,195 clusters each. From both trial 1 and trial 2, negative controls consisting of blank extractions carried through library preparation and enrichment were sequenced on separate individual Illumina MiSeq (2 × 250-bp) runs. After demultiplexing of the blank, all possible index combinations were retrieved to identify potential cross-contamination of libraries as well as exogenous bacterial contamination.

### Targeted capture of the stool sample.

Based on qPCR values and the average fragment sizes of each library generated from the human stool DNA extract, we combined various nanogram amounts of library (50, 100, 200 ng) and probes (25, 50, 100, 200, 400 ng) for enrichment (Table S3). Along with the negative-control (blank) library, we introduced additional negative controls during enrichment, using distilled H_2_O to replace the volume normally required for library input. We performed enrichment following the instructions in the myBaits manual (v4; Arbor Biosciences, Ann Arbor, MI) at a hybridization temperature of 65°C for 24 h. After hybridization and capture with Dynabeads (MyOne streptavidin C1 beads; catalog number 65001; Thermo Fisher, Waltham, MA), the resulting enriched library was amplified through 14 cycles of PCR using Kapa HiFi HotStart ReadyMix polymerase with library-nonspecific primers and the same conditions described above (see the enrichment methods in the supplemental material). The resulting products were purified using Kapa Pure beads (catalog number KK8000; Roche Canada) at a 1× volume ratio and eluted in 10 mM Tris, pH 8.0. Purified libraries were quantified through qPCR using 10× SYBR Select master mix (catalog number 4472942; Applied Biosystems, Foster City, CA) for Bio-Rad Cfx machines, Illumina specific primers (a 10× primer mix from Kapa; catalog number KK4809; Roche Canada), and Illumina’s PhiX control library (catalog number FC-110-3001; Illumina, San Diego, CA) as a standard. Cycling conditions were as follows: (i) 50°C for 2 min, (ii) 95°C for 2 min, (iii) 95°C for 15 s, (iv) 60°C for 30 s, and (v) a repeat of steps (iii) and (iv) for 40 cycles total. We pooled the enriched libraries in equimolar amounts based on qPCR values, and the McMaster Metagenomic Sequencing facility performed library quality control as described above. Finally, we sequenced the enriched libraries (average, 97,286 clusters) and the preenrichment libraries (average, 5,325,185 clusters) with an Illumina MiSeq sequencer (v2; 2 × 250 bp). The negative controls consisting of blank extractions carried through library preparation and enrichment were sequenced on separate individual Illumina MiSeq (2 × 250-bp) runs. After demultiplexing, all possible index combinations were retrieved.

### Analysis of bacterial isolate sequencing data.

In order to identify probe-targeted regions and coordinates that overlap predicted resistance genes based on RGI results for the individual bacterial genomes, we aligned our probe set to the draft reference genome sequence using the Bowtie2 (v2.3.4.1) program (47). We used the Skewer (v0.2.2) program (skewer -m pe -q 25 -Q 25) to trim sequencing reads (enriched or shotgun) and the bbmap (v37.93) program tool dedupe2.sh to remove duplicates and mapped the reads to the bacterial genomes using the Bowtie2 (v2.3.4.1) program (settings, –very-sensitive-local, unique sites only) (https://github.com/BioInfoTools/BBMap) (47, 56). Aligned reads were filtered based on mapping quality (≥41 MAPQ) and length (≥40 bp) using various tools: samtools (v1.4), bamtools (v2.4.1), and bedtools (v2.27.1) (48, 49, 57). We determined the number of reads mapping to the reference genome overall and the number of reads mapping within a predicted probe-targeted region using genomic coordinates and bedtools (intersectBed) (50). The percent length coverage and the average depth of coverage of each probe-targeted region with at least one read were determined using bedtools coverage (settings, -counts, -mean and default function) (49). We normalized the read counts by the number of reads mapping per kilobase of targeted region per the total number of reads mapping to a particular genome. The number of genes with at least 1, 10, or 100 reads was counted, and their percent length coverage by reads was determined.

### Analysis of stool sample sequencing data.

We processed the enriched and shotgun reads for the human stool sample as described above for the bacterial isolates. Subsampling of reads was performed using the seqtk (v1.2-r94) program (settings, seqtk sample -s100; https://github.com/lh3/seqtk). We used the bwt feature in RGI (the beta version of v5.0.0; http://github.com/arpcard/rgi) to map trimmed reads, using the Bowtie2 (v2.3.4.1) program, to the sequences in CARD (v3.0.0), generating alignments and results without any filters (47). We parsed the gene mapping and allele mapping files to determine the number of genes in CARD with reads mapping (at least 1, 10, and 100 reads) under various filters. After plotting the mapping quality for each read in every sample across the 3 sets, we chose an average mapping quality (mapq) filter of 11. We assessed a percent length coverage filter of a gene by reads of 10, 50, and 80% and chose the most permissive (10%) for comparison between the shotgun and enriched samples. These low thresholds were necessary for analyzing the shotgun data to obtain any reasonable results at all. Finally, we used a filter to check for the probes mapping to the reference sequences in most comparisons, except to identify genes in the shotgun samples that would not be captured by our probe set. We repeated the same analysis process for the negative-control (blank) libraries. In phase 2, set 1, there were very few reads associated with the blank library after enrichment, so we used the raw sequencing reads for analysis. For the blank in set 2, we omitted deduplication, and we could not identify any reads associated with the blank indexes after sequencing for set 3. Read counts were normalized using the all mapped reads column in the gene mapping file and the reference length (in kilobases) along with the total number of reads per kilobase per million (RPKM) available for mapping. Hierarchical clustering was performed using the Gene Cluster (v3.0) and Java Tree View (v1.1.6r4) (http://bonsai.hgc.jp/~mdehoon/software/cluster/software.htm) programs, log transformation, and clustering arrays with an uncentered correlation (Pearson) and average linkage. For rarefaction analysis, we first aligned trimmed reads against the sequences in CARD (v3.0.0) using the Bowtie2 program, followed by filtering for a mapping quality of ≥11 (47). This file, along with an annotation file for CARD, was analyzed with the AmrPlusPlus rarefaction analyzer (http://megares.meglab.org/amrplusplus) (58), with subsampling every 1% of total reads and a gene read length coverage of at least 10%. The average number of genes identified after rarefaction was plotted and fit to a logarithmic curve to allow for simplified extrapolation. We generated heat maps and figures in Prism (v8) software for macOS (GraphPad Software).

### Data availability.

Raw sequencing reads (FASTQ) for the IIDR clinical isolate collection bacterial isolate genome assembly were deposited in NCBI under BioProject accession number PRJNA532924. All metagenomic sequencing results, enriched or shotgun, were deposited in NCBI under BioProject accession number PRJNA540073. The probe set sequences and annotations are available at https://card.mcmaster.ca/download.

## Supplementary Material

Supplemental file 1
