# Supplementary material for: Capturing the Resistome: a Targeted Capture Method To Reveal Antibiotic Resistance Determinants in Metagenomes
Source: Antimicrob Agents Chemother. 2019 Dec 20;64(1):e01324-19. doi: 10.1128/AAC.01324-19 (PMC7187591; doi:10.1128/AAC.01324-19)
Supplement: Supplemental file 1 [file AAC.01324-19-s0001.pdf]

Supplementary methods: 2
Supplementary Figure 1: Probe and CARD nucleotide sequence statistics 5
Supplementary Table 1: Bacterial strains used in control experiments. 6
Supplementary Table 2: Library and sequencing information. 7
Supplementary Table 3: Individual strain enrichment results. 9
Supplementary Figure 2: Consistency in library prep methods and trials. 10
Supplementary Table 4: Pooling of genomic DNA to create "mock metagenomes" 11
Supplementary Table 5: Enrichment results to probe-targeted regions in pooled samples 13
Supplementary Figure 3: Sensitivity in recovery of bait-targeted regions with reads 16
Supplementary Figure 4: Enrichment results in higher read counts on antibiotic resistance genes compared to shotgun sequencing 17
Supplementary Table 6: Control enrichment with *Escherichia coli* C0002. 18
Supplementary Figure 5: Genes identified in positive control enrichments. 19
Supplementary Table 7: Phase 2 enrichment results with the full number of reads. 20
Supplementary Figure 6: Comparing enriched and shotgun ARG recovery. 21
Supplementary Table 8: Phase 2 enrichment results with subsampled reads. 21
Supplementary Table 9: Phase 2 overlapping genes with the full number of reads. 22
Supplementary Table 10: Phase 2 overlapping genes with subsampled reads. 22
Supplementary Figure 7: Hierarchical clustering of enriched libraries 24
Supplementary Figure 8: Hierarchical clustering of enriched and shotgun libraries 25
Supplementary Figure 9: Rarefaction curves for identification of antibiotic resistance genes. 26
Supplementary Table 11: Sequencing reads identified in the Blank samples. 27
Supplementary Table 12: Negative control enrichment with Blank samples 29
Supplementary Table 13: Genes identified through metagenomic analysis of enriched and shotgun samples. 29

Supplementary methods:
Modified Library Preparation for Trial 2

The same genomic DNA (except for *Pseudomonas aeruginosa* C00060) used in Trial 1 was used for library construction through a modified library preparation protocol (1). Briefly, ~ 200 nanograms of sonicated genomic DNA (250 - 300 bp) was used for library preparation at a volume of 25 µL. Deviations from the protocol include the use of KAPA Pure Beads (KK8000, Roche Canada, Laval, QC) instead of AMPure beads, oligonucleotides ordered from Integrated DNA Technologies (IDT, Coralville, IA, USA) (the ready-to-use adapter mix was diluted to 10uM of each adapter prior to use), and a total reaction volume of 50 µL for blunt end repair. Briefly, blunt end repair with T4 polynucleotide kinase (10U/µL) and T4 DNA polymerase (5U/µL) was carried out for 15 min at 25ºC and 15 min at 12ºC in a modified buffer (50mM NaCl, 10mM Tris-HCl, 10 mM $MgCl_2$, 100 µg/ml BSA, pH 7.9 at 25C) with 1 mM DTT, 100 µM dNTPs (2.5 mM each), and 1 mM ATP. Adapter ligation was performed using a final adapter concentration of 2.5µM, with T4 DNA ligase (0.125U/µL) in T4 DNA ligase buffer (1X) and PEG-4000 (5%) for 15 hours at 16ºC, then held at 4ºC. The reaction was purified using KAPA Pure beads at 1.8X and eluted in 20 µL of EBT (10 mM Tris-Cl, pH 8.3, 0.05% Tween-20). This was followed with adapter fill-in with Bst polymerase, large fragment (8U/µL) in ThermoPol reaction buffer (10X) with dNTPs (final concentration of 250 µM each) (30 mins at 37ºC; 80ºC for 20 mins; hold at 4ºC). Purification with library size-selection was performed using KAPA Purebeads (0.6X-0.8X ratio of beads to reaction volume) and products were eluted in 30 µL of 10mM Tris-HCl, pH 8.3. Indexing PCR was performed using 12.5 µL of template, KAPA HiFi Hotstart DNA polymerase ready mix (2X), (750nM of each indexing primer – 8 bp primers ordered from IDT diluted in nuclease free water at a stock concentration of 100µM) in a 40 µL reaction with the following conditions: 1) 98ºC for 45 sec; 2) 98ºC for 15 sec; 3) 60ºC for 45 sec; 4) 72ºC for 30 sec; 5) Repeat

2-4 for 12 cycles total; 6) 72ºC for 1 min. Libraries were purified with 1.5X KAPA Pure beads then eluted in 33 µL 10 mM Tris pH 8.0. Re-amplification of libraries for 2 cycles was performed using 1 µL of each library sample and KAPA HiFi Hotstart DNA polymerase ready mix (2X) and Library Amplification Primer Mix (10X) supplied by KAPA in the Library Amplification Kit with the followed conditions: 1) 98ºC for 45 sec; 2) 98ºC for 15 sec; 3) 60ºC for 45 sec; 4) 72ºC for 30 sec; 5) Repeat 2 - 4 for 2 cycles total; 6) 72ºC for 1 min. Libraries were purified using 1.5X Kapa Purebeads and eluted in 33 µL of 10 mM Tris-HCl pH 8.3. A negative control was also included consisting of a blank DNA extraction carried throughout the DNA extraction of bacterial DNA. Bioanalyzer and qPCR analyses were performed through the McMaster Farncombe Metagenomics Facility on all libraries including the blanks.

Enrichment methods

For Phase 1 Trial 1, the hybridization mix and blockers mix were prepared as described in the myBaits® manual version 3 (Arbor Biosciences, Ann Arbor, MI). 7 µL of DNA library (~100 ng) was added to the blockers mix and libraries were denatured at 95ºC for 5 minutes. The hybridization mix and libraries with blockers were brought to the hybridization temperature of 65ºC for 5 minutes. Hybridization mixes including the appropriate concentration of probeset were mixed with libraries and incubated at 65ºC for 16 hours. After 16 hours, the wash buffer was prepared and heated to 65ºC. Dynabeads MyOne Streptavidin C1 beads were prepared with binding buffer (10 mM Tris-HCl pH7.5, 1 mM EDTA, 2M NaCl in nuclease-free water) by three washes with binding buffer, and finally resuspended in binding buffer then heated to 65ºC (Thermo Fisher, Waltham, MA). Prepared beads were added to capture reactions, incubated at 65ºC for 30 minutes with frequent mixing, then pelleted on a magnetic rack. Beads were washed with pre-heated wash buffer (Supplied in the myBaits® kit), incubated for 10 minutes at 65ºC and the wash

was repeated two times. The beads were finally resuspended in 30 µL 10mM Tris-Cl, 0.05% Tween-20, pH 8.3. Post-capture amplification was performed on 15 µL of the bead-bound library with for 30 cycles of amplification using KAPA HiFi Hotstart DNA polymerase ready mix (2X), Library Amplification Primer Mix (10X) with the followed conditions: 1) 98ºC for 45 sec; 2) 98ºC for 15 sec; 3) 60ºC for 45 sec; 4) 72ºC for 30 sec; 5) Repeat 2-4 for 30 cycles total; 6) 72ºC for 1 min. Reactions were purified with KAPA Purebeads (0.8X cleanup) and eluted in 30 µL of 10 mM Tris-HCl, pH 8.3. These reaction products were purified with KAPA pure beads (1X) and eluted in 30 µL 10 mM Tris-HCl, pH 8.3. This is an over-amplified product and likely contains heterodimers of library fragments. An aliquot (2 µL) was used for an additional 3 cycles of amplification with the same primers and conditions as above. A 1X KAPA Purebeads purification was performed and products were eluted in 30 µL TE buffer. The concentration was verified by absorbance, followed by the Bioanalyzer and qPCR through the McMaster Metagenomics Facility.

For Trial 2, conditions were similar to the enrichment for Trial 1 libraries except for post-enrichment which was performed using 7.5 µL of enriched library, 12.5 µL of KAPA Ready Mix 2X, 1 µL of 10X primer mix (1) 98ºC 45 sec; 2) 98ºC 30sec; 3) 60ºC for 45sec; 4) 72ºC for 45sec; 5) Repeat step 2 - 4 for 17 cycles; 6) 72ºC for 5 min; 7) 4ºC hold. PCR products were purified using KAPA Pure beads (1.5X) and eluted in 10 mM Tris-HCl pH 8.0. The concentration was determined via absorbance and additional library quality control was performed through the McMaster Metagenomics Facility (Bioanalyzer and qPCR). Products were sequenced via Illumina MiSeq 2 x 250 bp.

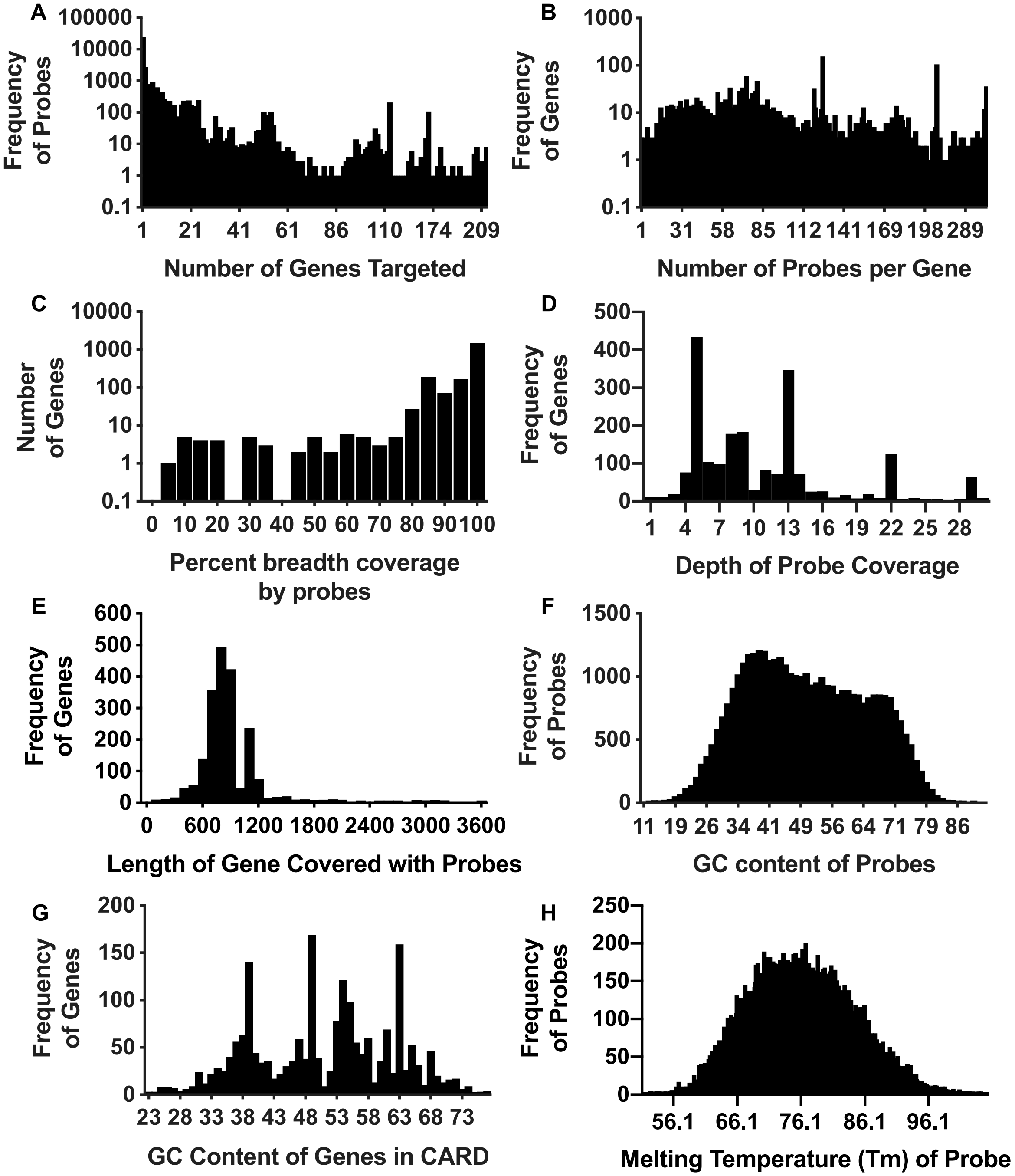

Supplementary Figure 1: Probe and CARD nucleotide sequence statistics

A) Number of genes targeted by probes through mapping with Bowtie2. B) Number of probes targeting genes through mapping with Bowtie2. C) Percent length coverage of genes with probes. D) Mean depth of probe coverage across individual genes in CARD. E) Length of sequence targeted by probes in genes in CARD. F) GC content of probes. G) GC content of genes in CARD. H) Melt temperature of final list of probes. Figures generated using Prism 8 for macOS.

Supplementary Table 1: Bacterial strains used in control experiments.

Clinical bacterial isolates obtained through the Wright Clinical Collection. Bacterial genomes were sequenced, and draft genome assemblies were analyzed through the Resistance Gene Identifier in CARD to predict the number of resistance genes. The total probeset was mapped against the draft assembled genome and the number of genes with probe coverage, percentage of genome covered by probes and overlap between predicted RGI genes and probe coverage were determined.

Bacterial strain	Genome size (Mb)	GC Content (%)	Predicted genes by RGI	Region predicted by RGI (%)	# Probe-targeted sites	Length of probe-targeted site (average and range)	Region with probe coverage (%)	RGI genes with probes	Region predicted by RGI and targeted by probes (%)
Escherichia coli C0002	5.29	50.62	67	1.64	65	797.75 (80 - 3595)	0.97	43	0.81
Klebsiella pneumoniae C0006	5.45	57.23	30	0.55	35	331.54 (80 - 877)	0.21	17	0.17
Staphylococcus aureus C0018	2.92	32.66	16	0.55	13	1127.54 (140 – 2013)	0.50	12	0.41
Staphylococcus aureus C0033	2.92	32.77	16	0.64	14	1143.07 (155 – 2130)	0.52	13	0.44
Klebsiella pneumoniae C0050	5.60	57.05	34	0.63	40	346.18 (80 – 900)	0.25	18	0.19
Pseudomonas aeruginosa C0060	6.80	66.19	53	1.18	48	933.35 (97 – 3415)	0.66	33	0.54
Escherichia coli C0094	5.22	50.74	67	1.65	64	779.86 (80 – 3003)	0.95	41	0.79
Pseudomonas aeruginosa C0292	6.81	66.21	54	1.17	48	938.71 (97 – 3415)	0.66	33	0.57

Supplementary Table 2: Library and sequencing information.
The amount in nanograms of each library and the corresponding amount of probes used for enrichment. The average size of library fragments prior to enrichment was determined through the Agilent Bioanalyzer 2100. The number of clusters (paired-end reads) that were generated for each library when sequenced by Illumina's MiSeq V2 2x250. Blanks for each trial were included and sequenced on a separate run; many of the blank libraries did not generate peaks on the Bioanalyzer nor any signal by quantitative PCR therefore their values are N/A. In Phase 2, three positive controls for enrichment were included with genomic DNA from *Escherichia coli* C0002 and varying library and probe amounts.

Phase	Trial/Set	Library	Amount of Probes (ng)	Amount of Library (ng)	Average Library Size (bp)	Clusters sequenced enriched	Clusters sequenced shotgun
Phase 1	Trial 1	C0002	100	100	988	66926	
		C0018	100	100	994	75860	
		C0050	100	100	1222	73941	
		C0060	100	100	1225	81810	
		Pool 1	100	100	1257	61568	218008
		Pool 2	100	100	1158	61658	159059
		Pool 3	100	100	1216	58308	109194
		Negative Control - Blank	100	N/A	632	170565	
	Trial 2	C0002	100	100	435	99748	
		C0018	100	100	438	143804	
		C0050	100	100	416	153673	
		C0060	100	100	403	124971	
		Pool 1	100	100	429	86023	29241
		Pool 2	100	100	413	124170	33488
		Pool 3	100	100	427	127682	32560
		Negative Control - Blank	100	N/A	345	44026	
Phase 2	Set 1	1 - 1	25	50	952	89768	
		1 - 2	50	50	968	77117	
		1 - 3	100	50	919	65746	
		1 - 4	50	100	1044	55783	
		1 – 5	100	100	972	64761	
		1 - 6	200	100	940	71099	3652948
		1 - 7	100	200	915	15211	4405779
		1 - 8	200	200	1020	59409	
		1 - 9	400	200	998	25911	
		Negative Control - Blank	50	N/A	276	2590	

Positive Controls	C0002 – 1 - 1	100	50	986	80647	
	C0002 – 1 - 2	50	50	939	116965	
	C0002 – 1 - 3	25	50	976	112881	
Set 2	2 - 1	25	50	955	158710	
	2 - 2	50	50	887	100590	
	2 - 3	100	50	891	102689	
	2 - 4	50	100	902	120764	
	2 - 5	100	100	956	141994	6151998
	2 - 6	200	100	941	159192	
	2 - 7	100	200	790	96211	
	2 - 8	200	200	944	129333	
	2 - 9	400	200	871	76195	7660355
	Negative Control - Blank	50	N/A	N/A	3804	
Positive Controls	C0002 – 2 -1	100	33	993	139909	
	C0002 – 2 - 2	50	50	935	235429	
	C0002 – 2 - 3	25	50	876	129070	
Set 3	3 - 1	25	50	854	82778	5866495
	3 - 2	50	50	888	158968	
	3 - 3	100	50	910	65675	
	3 - 4	50	100	889	103671	
	3 - 5	100	100	882	78251	4213540
	3 - 6	200	100	943	68331	
	3 - 7	100	200	820	96722	
	3 - 8	200	200	934	79036	
	3 - 9	400	200	917	82375	
	Negative Control - Blank	50	N/A	N/A	5962	
Positive Controls	C0002 – 3 -1	100	38	846	54117	
	C0002 – 3 - 2	50	32	881	96258	
	C0002 – 3 - 3	25	38	779	110746	

Supplementary Table 3: Individual strain enrichment results.
Strains were enriched individually in two trials with different library sizes. For each strain the regions predicted to be targeted by probes were determined through mapping the probeset to each individual genome). Enrichment results across two trials were determined by mapping trimmed and filtered reads to genome, calculating the percentage on-target and normalizing reads and depth per kb per million reads for probe-targeted regions with at least 10 reads.

Strain	Average % mapping to genome	Average % mapping to probe-targeted sites	% of targeted regions with at least 10 reads	Average % coverage of targeted regions	Average reads per kb per million reads on probe-targeted region	Average depth per kb per million reads on probe-targeted region
Escherichia coli C0002	96.67 (±2.72)	95.07 (±1.54)	100	100	18975.73 (±414.91)	6192.13 (±297.27)
Staphylococcus aureus C0018	97.99 (±1.98)	94.89 (±2.31)	100	100	67615.06 (±4360.20)	19968.28 (±2670.37)
Klebsiella pneumoniae C0050	95.60 (±3.96)	85.74 (±4.68)	100	100	40531.43 (±2516.77)	17315.24 (±1630.66)
Pseudomonas aeruginosa C0060	91.45 (±5.49)	90.73 (±0.95)	100	100	22725.67 (±32.97)	6497.48 (±61.46)

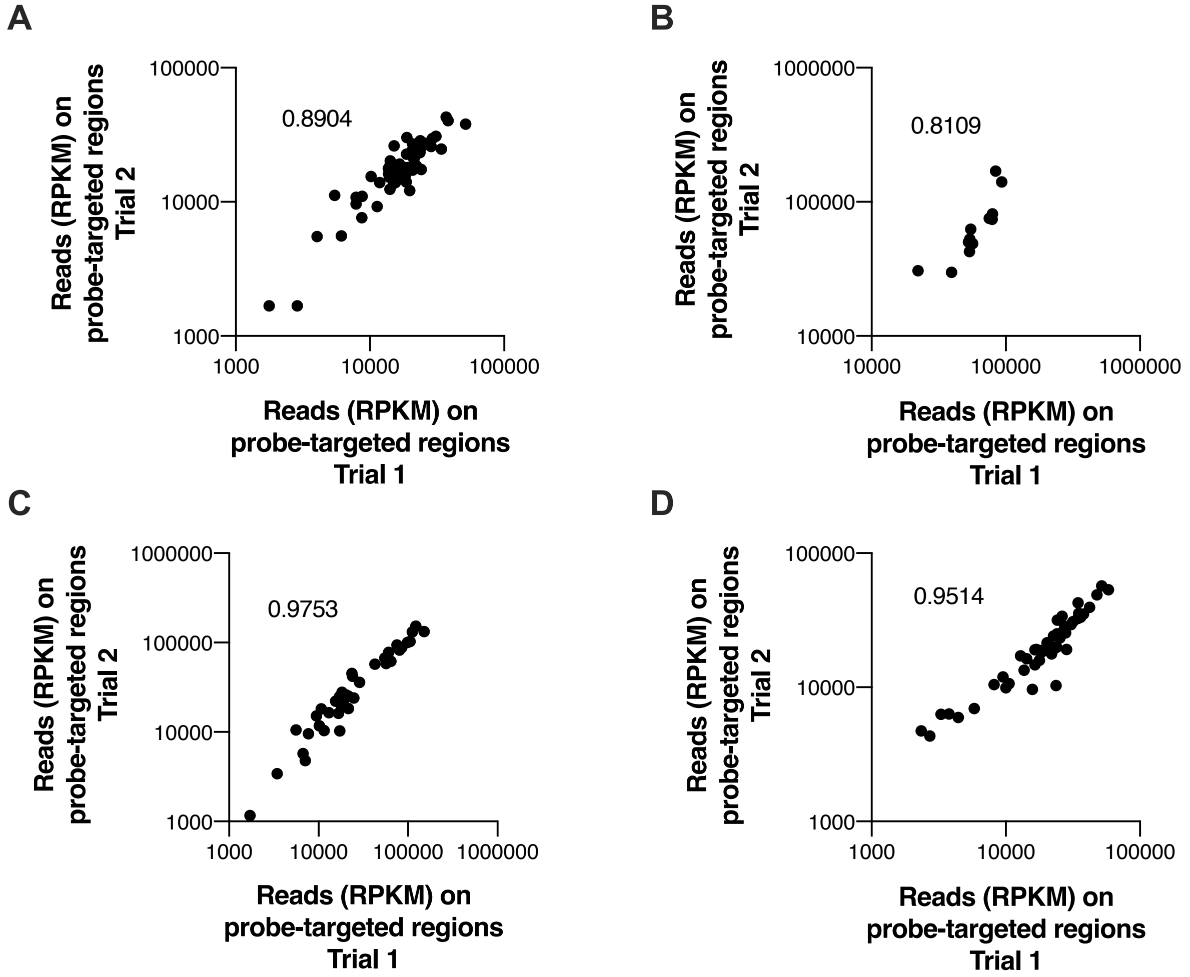

Supplementary Figure 2: Consistency in library prep methods and trials.
Comparing read counts normalized in subsampled individual enrichment trials through different library prep methods. Reads from enrichment of individual genomes of *Escherichia coli* C0002 (A), *Staphylococcus aureus* C0018 (B), *Klebsiella pneumoniae* C0050 (C) and *Pseudomonas aeruginosa* C0060 (D) in Trial 2 were subsampled to same depth as reads in Trial 1. The reads were mapped to the respective bacterial genome, filtered for mapping quality and then the number of reads on each RGI and probe-targeted region were counted and normalized per kb per million reads. Pearson correlation coefficients are shown.

Supplementary Table 4: Pooling of genomic DNA to create "mock metagenomes"

We pooled various nanogram amounts of genomic DNA from bacteria and estimated the percentage of each strain in the respective pools based on total genome size of each strain. With reads generated through shotgun sequencing and after enrichment, we calculated the percentage of reads mapping to a particular genome by mapping to a combined reference of the genomes used in a given pool and counting the reads that mapped to each respective genome (= reads mapping to genome A / reads mapping to all genomes). The percentage represented by targeted regions of each genome in each pool is determined by taking the percentage of each isolate's genome that is targeted by probes and the percentage of reads that map to that particular genome from the shotgun sequencing data.

Pool	Strain	Amount of genomic DNA pooled (ng)	Estimated % of pool	% of reads mapping from shotgun	Targeted % of pool	% of reads mapping from enriched
Trial 1 Pool 1	C0002	312	21.98	24.82	0.24	52.55
	C0018	312	40.00	12.06	0.060	32.12
	C0050	312	20.74	27.18	0.068	8.86
	C0060	312	17.28	35.93	0.24	6.47
Trial 2 Pool 1	C0002	112	18.77	22.30	0.22	33.95
	C0018	174	53.01	65.29	0.33	62.88
	C0050	106	16.79	4.39	0.011	1.54
	C0060	88	11.43	8.02	0.053	1.63
Trial 1 Pool 2	C0002	1250	66.30	64.73	0.63	71.26
	C0018	180	17.22	11.96	0.060	19.69
	C0050	180	9.07	11.28	0.028	4.75
	C0060	180	7.41	12.03	0.079	4.30
Trial 2 Pool 2	C0002	264	48.04	57.31	0.56	65.39
	C0018	102	33.92	35.54	0.18	33.24
	C0050	62	10.75	1.66	0.0042	0.44
	C0060	51	7.29	5.49	0.036	0.94
Trial 1 Pool 3	C0002	125	11.01	13.91	0.13	38.50
	C0006	125	10.70	24.75	0.052	2.34
	C0018	125	19.88	6.54	0.033	11.62
	C0033	125	19.88	11.59	0.060	22.81
	C0050	125	10.40	12.75	0.032	2.73
	C0060	125	8.56	16.40	0.11	2.16
	C0094	125	11.01	6.90	0.066	18.78
	C0292	125	8.56	7.15	0.047	1.07
Trial 2 Pool 3	C0002	46	8.65	9.84	0.095	14.80
	C0006	83	8.16	14.44	0.030	1.53
	C0018	43	28.17	11.49	0.057	12.49
	C0033	36	28.15	34.36	0.18	34.58
	C0050	45	7.68	0.60	0.0015	0.13
	C0060	83	5.20	2.02	0.013	0.42
	C0094	46	8.78	25.21	0.24	35.67
	C0292	36	5.21	2.04	0.013	0.39

Supplementary Table 5: Enrichment results to probe-targeted regions in pooled samples

Genomic DNA from individual strains was pooled in various ratios to produce “mock metagenomes” for enrichment. For each strain, the regions predicted be targeted by probes (determined through mapping the probeset to each individual genome) are considered the targeted region for analysis. Trimmed and filtered reads from paired enriched and shotgun pools were subsampled to same read depth. The resulting reads were mapped to the individual strain’s genomes, counted on-target and normalized per kb per million reads mapping. Percentage on-target, percentage of probe-targeted regions with at least 10 reads as well as their percent coverage, average reads, and average depth were determined for each strain at the probe-targeted region level. The fold enrichment is based on all genes regardless of read counts.

Sample	Strain	% of reads in Pool	% Mapping to probe-targeted regions	% of probe-targeted regions with reads	% coverage of probe-targeted regions	Average reads per kb per million reads	Average depth per kb per million reads	Fold-enrichment in reads (average and range)
Trial 1 Pool 1 Enriched	C0002	52.75	93.06	100	100	19097.95	6091.42	810.18 (2.66 – 16590.95)
	C0018	20.05	94.84	100	100	67393.09	19715.42	135.84 (31.11 – 291.78)
	C0050	18.73	85.44	90	100	41944.82	16304.97	1341.88 (3.77 – 23020.26)
	C0060	3.40	90.26	91.67	98.73	24920.46	6697.48	994.87 (0 – 21945.61)
Trial 1 Pool 1 Shotgun	C0002	21.61	1.56	18.46	90.13	671.09	153.52	
	C0018	10.32	0.70	15.38	88.03	820.59	161.15	
	C0050	23.56	0.82	25.00	100	762.34	190.87	
	C0060	28.70	0.81	12.50	84.54	301.55	44.92	
Trial 2 Pool 1 Enriched	C0002	35.84	98.90	96.92	100	20081.94	6630.47	4972.95 (2.84 – 35942.31)
	C0018	56.55	98.56	100	100	74814.49	24542.74	144.41 (41.36 – 332.17)
	C0050	7.72	97.63	47.50	99.75	74609.44	24141.06	18991.42 (0 – 170582.07)
	C0060	1.31	93.37	47.92	83.22	30865.24	7310.50	17166.87 (0 – 70414.91)
Trial 2 Pool 1 Shotgun	C0002	23.52	1.49	1.54	91.65	471.34	30.86	
	C0018	57.30	0.71	76.92	79.03	570.56	98.30	
	C0050	5.19	0.88	0	0	0	0	
	C0060	6.65	0.65	0	0	0	0	
Trial 1 Pool 2 Enriched	C0002	68.39	77.35	96.92	100	15928.54	4982.54	57.09 (2.57 – 192.18)
	C0018	12.69	79.11	100	100	56570.38	16316.11	2614.81 (15.93 – 32565.71)
	C0050	12.61	74.13	75.00	99.93	41711.08	15702.37	2727.71 (0 – 39495.86)
	C0060	2.34	38.95	70.83	96.27	11523.24	2820.94	2382.94 (0 – 19387.19)
Trial 1 Pool 2 Shotgun	C0002	58.69	1.34	58.46	96.92	321.15	81.43	
	C0018	10.64	0.74	30.77	78.51	896.24	141.82	
	C0050	11.48	1.33	20	100	1745.41	464.15	
	C0060	9.72	0.75	2.08	56.38	266.69	18.15	

Trial 2 Pool 2 Enriched	C0002	65.64	98.29	96.92	100	19970.52	6708.67	1190.08 (7.74 – 29085.20)
	C0018	28.13	98.15	100	100	75034.93	24899.52	210.58 (32.41 – 596.02)
	C0050	10.26	98.23	47.50	100	77537.34	26906.17	8270.19 (0 – 50937.25)
	C0060	0.73	88.86	27.08	78.56	37440.00	8936.77	18933.20 (0 – 106732.35)
Trial 2 Pool 2 Shotgun	C0002	56.47	1.38	20.00	73.49	404.35	72.86	
	C0018	29.19	0.57	23.08	73.76	698.55	125.73	
	C0050	3.01	4.51	2.50	79.03	10409.44	2093.37	
	C0060	4.27	0.73	0	0	0	0	
Trial 1 Pool 3 Enriched	C0002	38.74	94.12	98.46	100	19755.27	6312.06	2493.04 (3.05 – 22767.27)
	C0006	13.66	84.08	91.43	100	51010.68	22066.06	3295.94 (0 – 61249.67)
	C0018	29.65	95.22	100	100	63154.77	15991.26	2909.12 (54.61 – 35638.08)
	C0033	33.17	94.82	100	100	56232.72	13178.66	156.78 (28.17 – 314.91)
	C0050	14.84	85.22	92.5	100	43478.45	18486.32	2475.78 (4.87 – 47799.65)
	C0060	2.45	91.97	89.58	98.78	26022.10	7430.52	3742.84 (3.65 – 62302.44)
	C0094	35.52	92.59	98.44	100	19949.59	6561.88	3526.16 (2.48 – 23220.26)
	C0292	2.78	84.96	91.67	99.29	28432.58	10574.24	4014.72 (0 – 54962.31)
Trial 1 Pool 3 Shotgun	C0002	9.83	1.63	3.08	88.69	1449.60	308.97	
	C0006	25.19	0.36	8.57	95.63	3450.36	1206.18	
	C0018	11.94	0.51	7.69	68.26	413.96	50.49	
	C0033	12.81	0.59	7.14	68.25	424.67	47.08	
	C0050	24.09	0.48	12.5	93.25	853.91	300.04	
	C0060	17.84	0.90	4.17	64.69	222.28	16.28	
	C0094	8.25	1.67	3.125	88.69	1726.91	368.08	
	C0292	16.78	0.94	4.17	64.69	1141.24	84.87	
Trial 2 Pool 3 Enriched	C0002	32.65	98.09	96.92	99.97	20307.57	6847.06	7369.15 (4.14 – 66339.3)
	C0006	7.75	90.49	51.43	99.50	86220.71	36708.00	25683.46 (0 – 271673.69)
	C0018	45.46	97.45	100	100	65485.29	17173.26	5819.09 (29.42 – 74023.04)
	C0033	52.11	97.53	100	100	58846.80	13719.18	698.58 (72.34 – 8084.37)
	C0050	8.22	92.65	50.00	99.55	74207.10	29767.85	21813 (0 – 256173.72)
	C0060	0.86	90.00	27.08	79.68	39544.66	8226.37	16172.91 (0 – 70505.29)
	C0094	34.91	97.65	96.87	100	20612.44	7021.48	7479.75 (2.67 – 61794.38)
	C0292	0.89	89.30	29.17	80.95	44281.92	13985.84	18128.93 (0 – 120321.02)
Trial 2 Pool 3 Shotgun	C0002	16.88	1.38	0	0	0	0	
	C0006	15.36	0.47	0	0	0	0	
	C0018	41.07	0.70	38.46	73.84	525.28	55.49	
	C0033	44.54	0.79	50.00	77.22	703.43	113.13	
	C0050	12.76	0.64	0	0	0	0	

C0060	4.54	0.77	0	0	0	0
C0094	21.50	1.23	1.56	68.13	404.24	25.04
C0292	4.59	0.86	0	0	0	0

U

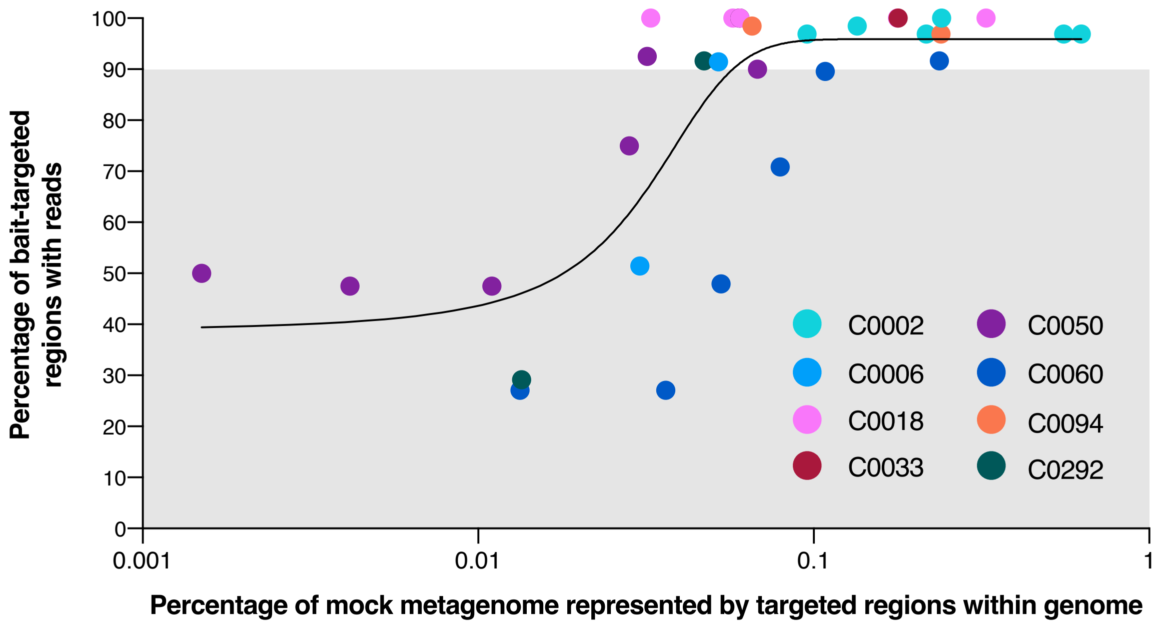

Supplementary Figure 3

Supplementary Figure 3: Sensitivity in recovery of bait-targeted regions with reads. Using the estimated percentage of a pool represented by each isolate's genome from the percentage of reads mapping in the shotgun dataset, the estimate percentage of the mock metagenome represented by that isolate's bait-targeted complement was determined. This represents the limit of detection or sensitivity for each isolate/genome tested. The Y-axis is the percentage of probe-targeted regions of a given isolate's genome that were successfully captured/recovered with at least 10 reads after filtering.

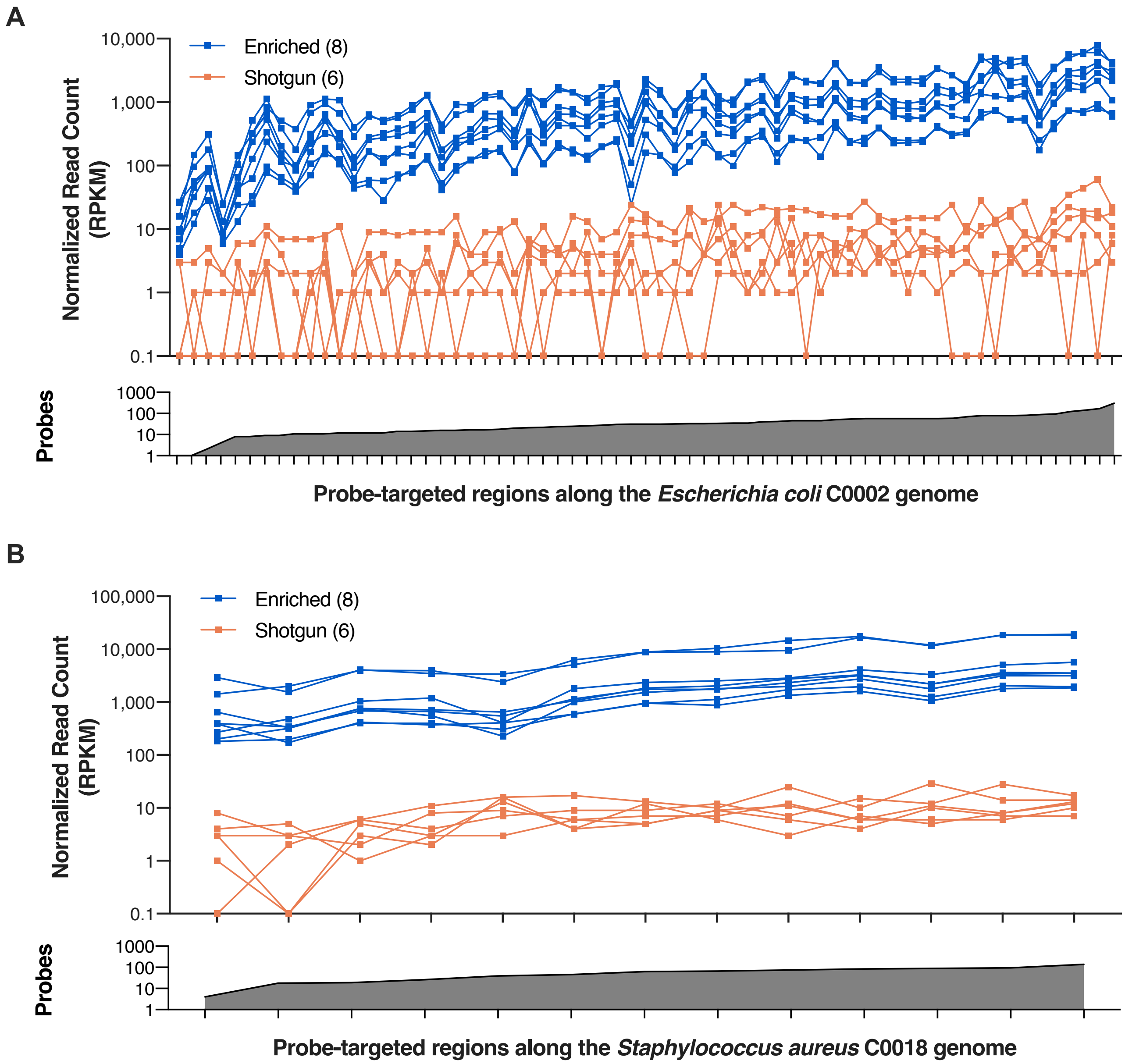

Supplementary Figure 4: Enrichment results in higher read counts on antibiotic resistance genes compared to shotgun sequencing. Raw read counts at each probe-targeted region within the *Escherichia coli* C0002 genome (**A**) and *Staphylococcus aureus* C0018 genome (**B**) in enriched and shotgun samples including individual and "mock metagenomes" of multiple strains. Among enriched and shotgun pairs, reads were subsampled to equal depths and mapped to the individual strain's genome. The predicted number of probes for each region along the genome are shown in the panels below. The Y axes are in the logarithmic scale.

Supplementary Table 6: Control enrichment with *Escherichia coli* C0002.
Enrichment results from the positive control of *E. coli* C0002 control used in Phase 2. Trimmed and deduplicated reads were mapped to CARD using RGIBWT, filtered by genes with probe coverage, an average read mapping quality >=11, and percent length coverage of a gene with reads >=80%.

	Probes (ng)	Library (ng)	% reads mapping to CARD	Total number of genes	Genes with map quality >=11	Genes with probes	Genes with length coverage with reads >=80%	Genes with probes and map quality >=11	Genes passing all filters
C0002 – Set 1	25	50	63.52	164	51	53	86	39	36
	50	50	64.81	164	54	53	84	39	36
	100	50	63.75	154	53	53	80	40	36
C0002 – Set2	25	50	61.10	179	62	54	82	42	36
	50	50	65.77	195	60	59	84	44	36
	100	33	60.31	170	59	57	87	42	36
C0002 – Set 3	25	38	65.46	182	58	57	86	39	36
	50	32	65.77	172	58	53	88	40	36
	100	38	67.98	147	54	56	83	42	36

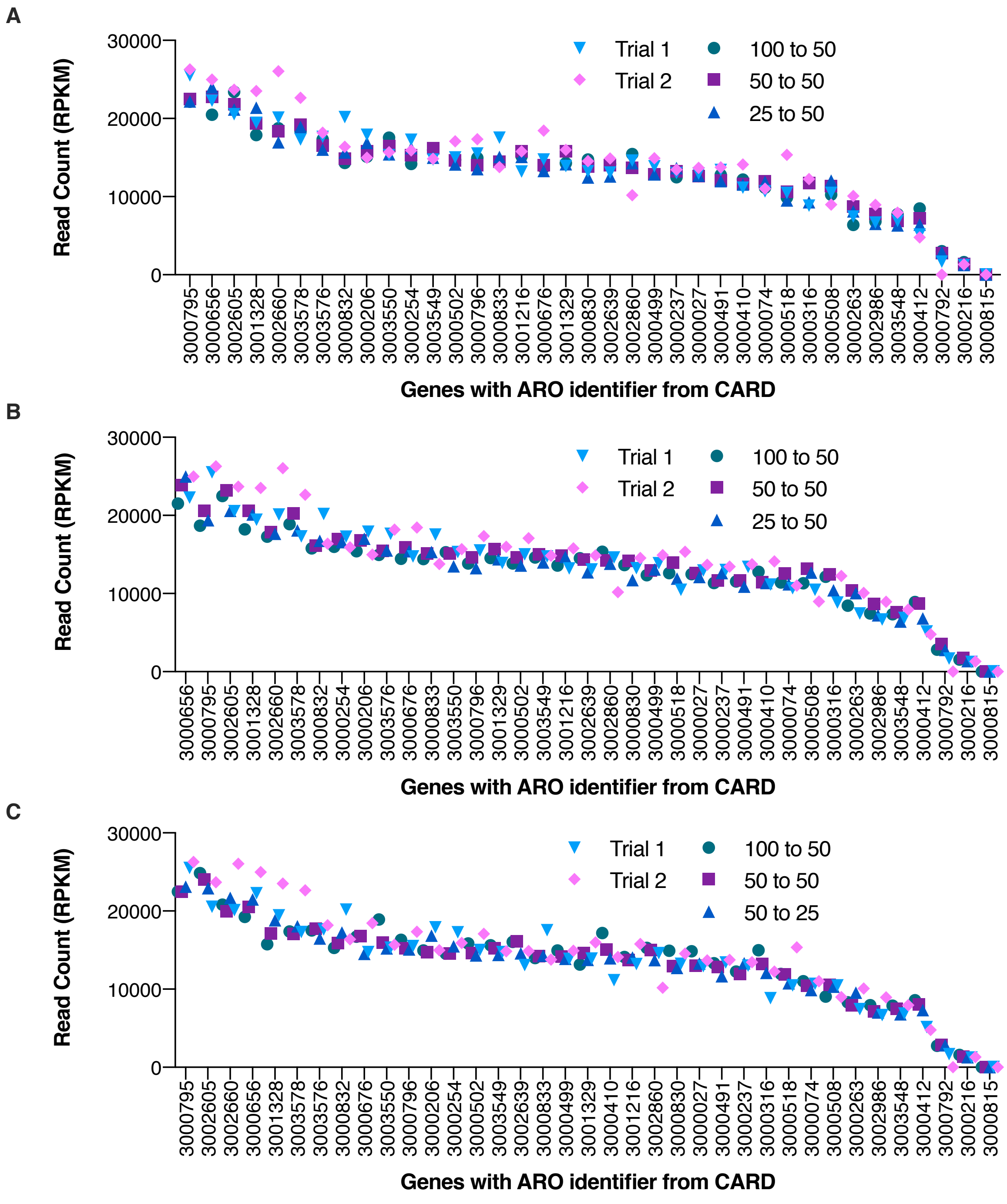

Supplementary Figure 5: Genes identified in positive control enrichments.
Normalized read counts from C0002 control enrichments from *rgi bwt* results from 3 samples in each Set (A) Set 1, B) Set 2, C) Set 3) to the two trials of individual enrichment. Genes with reads were filtered based on read mapping quality greater than or equal to 11, percent length coverage of a gene with reads greater than or equal to 80% and genes with probes mapping. Reads were normalized per kb of reference gene per million reads mapping. Genes are ordered by sum of read counts from highest to lowers (left to right) with the ARO identifier shown along the X axis

Supplementary Table 7: Phase 2 enrichment results with the full number of reads.
For the enriched samples, trimmed and deduplicated reads were mapped to CARD using RGIBWT, filtered by genes with at least 10 reads, those with probes, an average read mapping quality >=11, and length coverage of a gene with reads >=10%. For the shotgun samples, trimmed and deduplicated reads were mapped to CARD using RGIBWT, filtered by genes with an average read mapping quality >=11 and read length coverage of a gene >=10%. EN = enriched, UN = shotgun.

	Probes (ng)	Library (ng)	Reads mapping to CARD (%)	Total number of genes	Genes with read map quality >=11	Genes with probes	Genes with read length coverage >=10%	Genes passing all filters
**Sample Set 1**								
EN	25	50	55.36	60	50	51	58	48
	50	50	65.73	62	54	52	60	49
	100	50	55.59	60	50	50	60	48
	50	100	65.63	56	47	46	55	43
	100	100	51.85	61	51	51	60	48
	200	100	58.21	64	56	53	61	49
	100	200	51.52	34	26	27	34	25
	200	200	66.57	60	50	48	59	45
	400	200	49.44	45	37	36	43	33
UN	200	100	0.030	26	19	N/A	24	18
	100	200	0.030	32	22	N/A	29	20
**Sample Set 2**								
EN	25	50	64.07	78	67	64	76	61
	50	50	64.60	72	64	61	71	58
	100	50	57.96	75	64	61	74	57
	50	100	46.75	78	66	66	76	62
	100	100	58.99	79	69	64	77	61
	200	100	44.52	85	72	69	80	63
	100	200	60.43	76	66	62	73	59
	200	200	47.27	82	71	67	81	64
	400	200	41.22	70	59	58	69	55
UN	400	200	0.016	41	28	N/A	37	27
	100	100	0.032	34	24	N/A	32	23
**Sample Set 3**								
EN	25	50	50.16	72	63	61	70	58
	50	50	38.19	79	66	64	76	60
	100	50	51.73	69	59	59	68	55
	50	100	29.46	78	66	63	76	60
	100	100	40.28	74	65	60	72	57
	200	100	39.06	67	57	57	67	53
	100	200	29.97	69	57	58	68	54
	200	200	40.32	72	60	58	71	55
	400	200	43.74	69	58	56	67	53
UN	100	100	0.031	29	19	N/A	26	19
	25	50	0.031	34	23	N/A	30	22

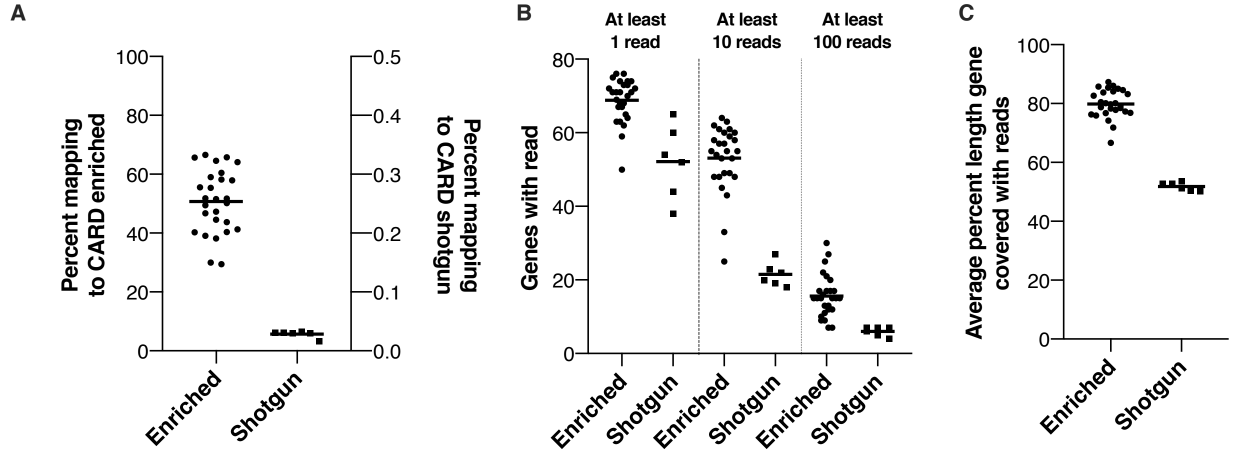

Supplementary Figure 6: Comparing enriched and shotgun ARG recovery. For the enriched and shotgun samples, the full number of reads for each sample were mapped to CARD using *rgi bwt*. A) The percentage of reads mapping to CARD. B) Genes were counted with at least 1, 10 and 100 reads and filtered for mapping quality (>=11), percent coverage by reads (>=10) and probes mapping (only for the enriched samples). C) The average percent coverage of all genes with at least 10 reads in each sample after the same filters used in B.

Supplementary Table 8: Phase 2 enrichment results with subsampled reads.
For the enriched samples, reads were subsampled to 22,324 reads and mapped to CARD using RGIBWT. Results were filtered by genes with at least 10 reads, those with probes, an average read mapping quality >=11, and length coverage of a gene with reads >=10%. For the shotgun samples, reads were subsampled to their paired enriched sample and mapped to CARD using RGIBWT. Results were filtered by genes with an average read mapping quality >=11 and read length coverage of a gene >=10%. EN = enriched, UN = shotgun.

	Probes (ng)	Library (ng)	Reads mapping to CARD (%)	Total number of genes	Genes with read map quality >=11	Genes with probes	Genes with read length coverage >=10%	Genes passing all filters
**Sample Set 1**								
EN	25	50	55.24	34	26	27	34	25
	50	50	65.84	39	31	31	37	28
	100	50	56.11	46	37	37	45	34
	50	100	66.01	39	32	32	39	30
	100	100	51.94	40	32	32	37	28
	200	100	57.93	38	30	30	37	28
	100	200	51.52	34	26	27	34	25
	200	200	66.99	42	34	33	39	30
	400	200	49.39	33	26	26	33	24
UN	200	100	0.038	2	2	N/A	2	2
	100	200	0.054	0	0	N/A	0	0
**Sample Set 2**								
EN	25	50	64.25	41	33	34	40	32

	50	50	64.11	43	36	35	40	31
	100	50	58.80	43	36	35	43	33
	50	100	46.95	40	32	33	38	29
	100	100	59.13	42	35	34	41	31
	200	100	44.64	45	35	34	41	31
	100	200	60.55	50	42	42	49	39
	200	200	47.29	45	38	37	45	35
	400	200	41.56	43	34	35	41	32
UN	400	200	0.029	1	1	N/A	1	1
	100	100	0.035	2	2	N/A	2	2
**Sample Set 3**								
EN	25	50	50.64	37	29	30	36	27
	50	50	37.85	27	19	20	27	18
	100	50	51.41	36	27	28	33	24
	50	100	29.56	29	21	22	28	20
	100	100	40.77	34	26	26	33	24
	200	100	38.86	37	30	30	37	28
	100	200	30.08	31	23	24	30	21
	200	200	40.62	34	26	26	32	23
	400	200	44.35	37	30	29	35	26
UN	100	100	0.023	0	0	N/A	0	0
	25	50	0.023	1	1	N/A	1	1

Supplementary Table 9: Phase 2 overlapping genes with the full number of reads.
We calculated the overlap of genes with at least 10 reads passing the percent length coverage by reads (>=10%), average read mapping quality (>=11) and probe mapping (except for shotgun libraries) filters.

Samples	Total genes	Genes found in all	Genes found in 2/3 or more	Genes found in 1/3 or more	Overlap in All Samples (%)
Set 1 Enriched	62	24	38	53	38.71
Set 2 Enriched	68	50	57	64	73.53
Set 3 Enriched	70	41	53	60	58.57
All Enriched	70	24	52	60	34.28
All Shotgun	32	16	18	28	50.00

Supplementary Table 10: Phase 2 overlapping genes with subsampled reads.
Libraries were subsampled to the same number of reads within sets and overall (22,324 reads). Shotgun libraries were subsampled to the same number of reads as the lowest enriched library overall. Resulting genes with at least 10 reads were filtered for percent coverage by reads (>=10%), average mapping quality (>=11) and probe mapping (except for the shotgun samples).

Samples	Total genes	Genes found in all	Genes found in 2/3 or more	Genes found in 1/3 or more	Overlap in All Samples (%)
Set 1 Enriched	38	16	26	32	42.10
Set 2 Enriched	45	22	30	36	48.89
Set 3 Enriched	37	13	20	26	35.14

All Enriched	47	13	24	31	27.66
All Shotgun	2	0	1	2	0

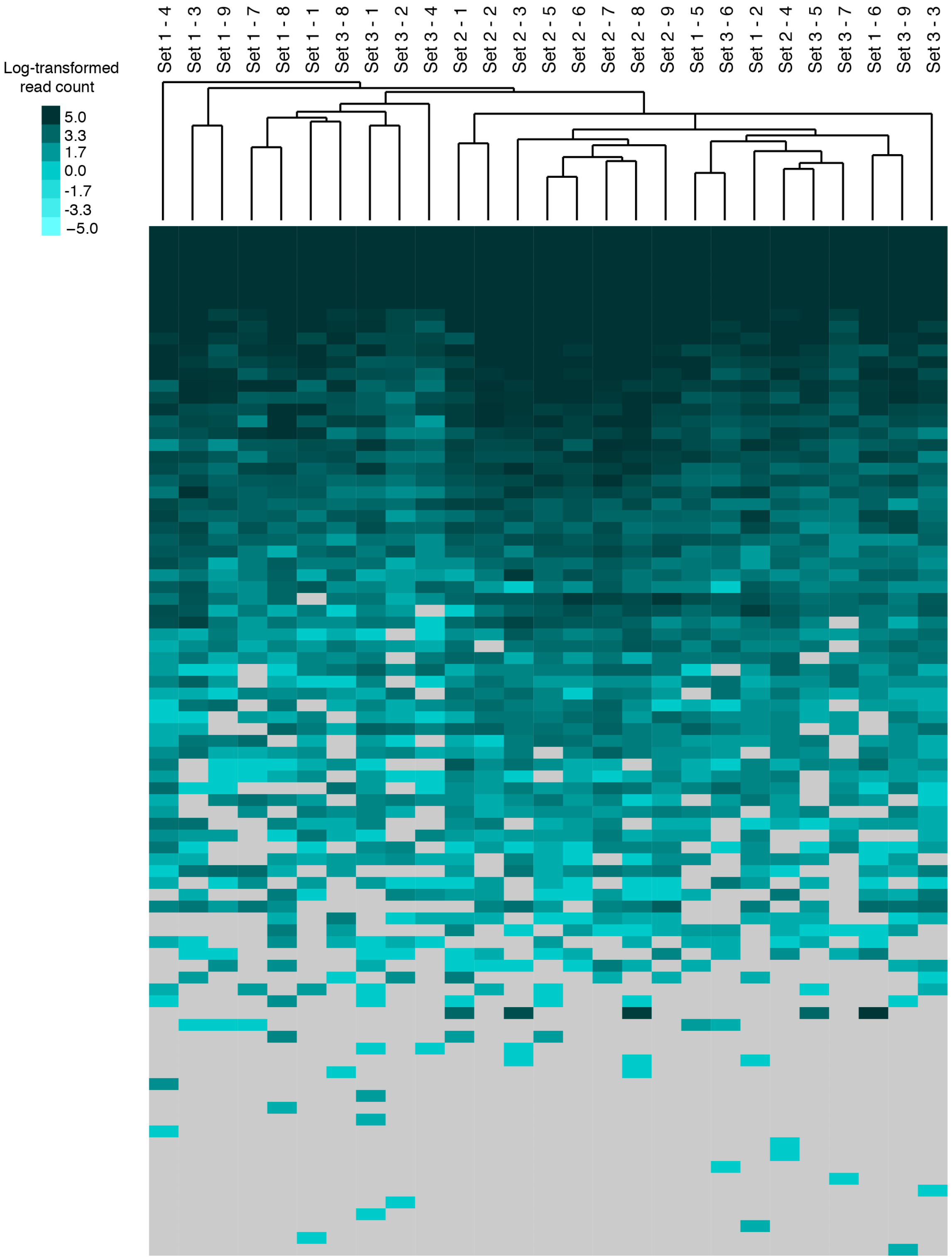

Supplementary Figure 7: Hierarchical clustering of enriched libraries. Enriched reads from 27 libraries were subsampled to 22,324 reads, mapped to CARD through *rgi bwt*. The reads were mapped to CARD through *rgi bwt* and filtered for genes with probes mapping, with greater than or equal to 10% length coverage by reads and an average read mapping quality >=11. Read counts were log-transformed and combined into a heatmap ordered by average read counts across the 27 enriched samples.

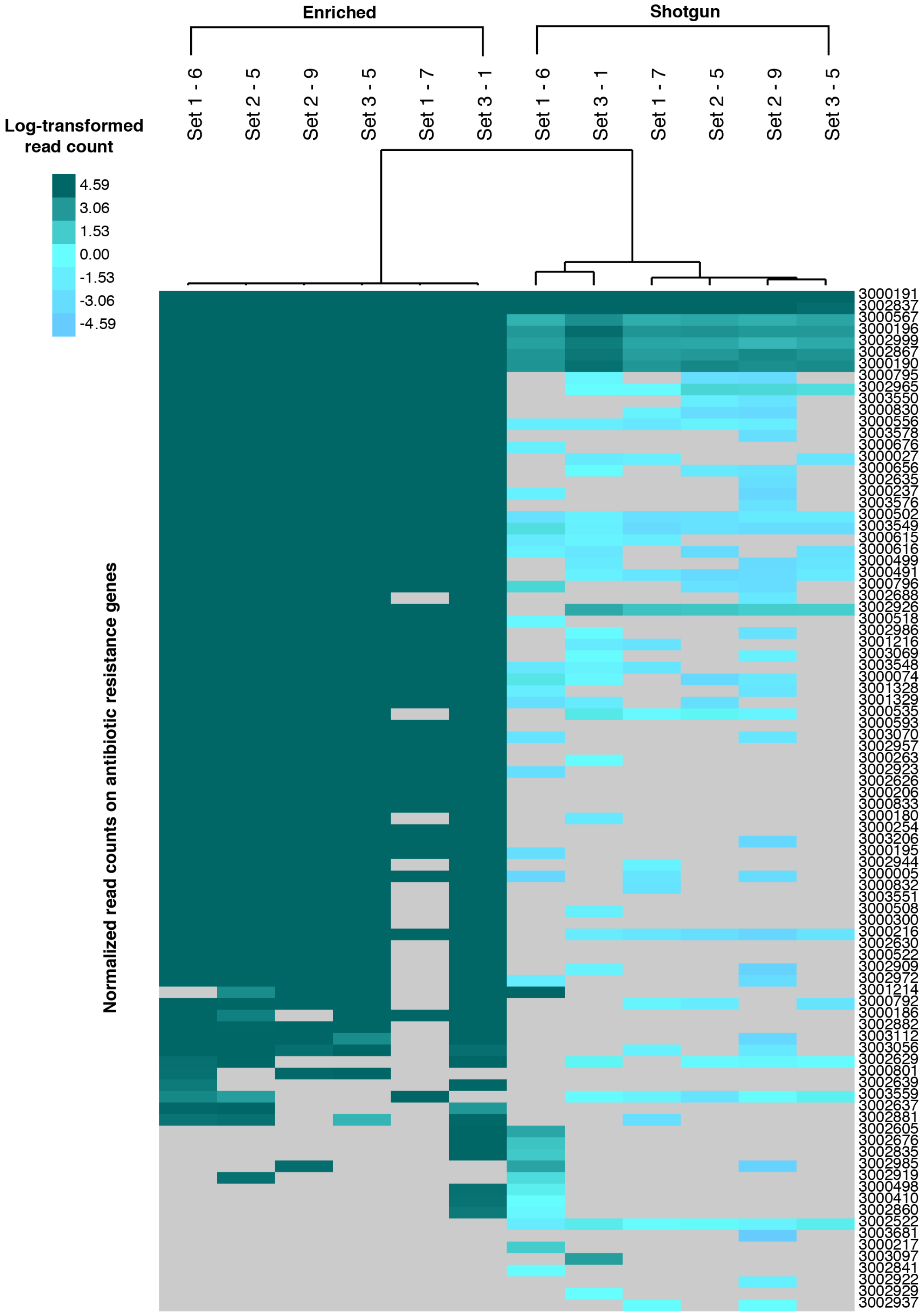

Supplementary Figure 8: Hierarchical clustering of enriched and shotgun libraries.
The full number of reads from the 6 enriched and shotgun pairs were mapped to CARD through *rgi bwt*. The results were filtered for genes with greater than or equal to 10% read length coverage and an average read mapping quality greater >= 11. Read counts were normalized by kb of gene and reads available for mapping, log-transformed, and combined into a heatmap. Genes are

ordered by sum of read counts. ARO numbers from CARD are shown on the right-hand side of the heatmap.

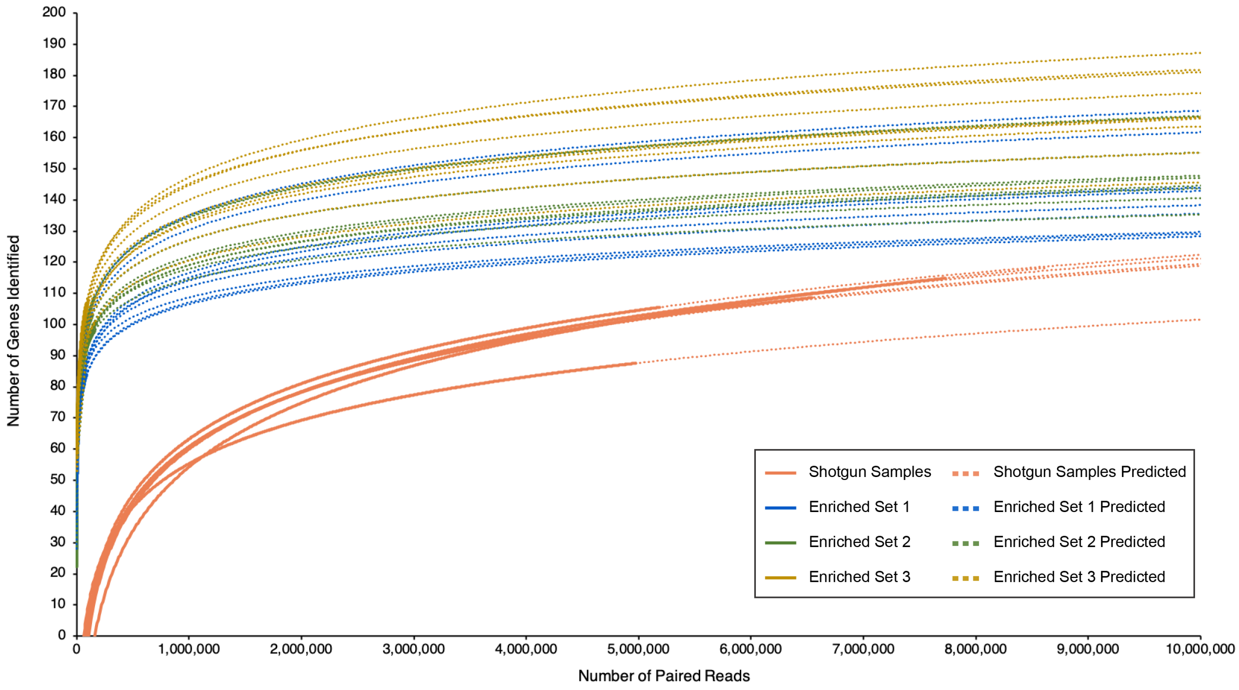

Supplementary Figure 9: Rarefaction curves for identification of antibiotic resistance genes. The AmrPlusPlus Rarefaction Analyzer was used with subsampling every 1% of the total reads and a gene read length of at least 10% to identify antibiotic resistance genes. The solid lines show individual sequencing experiments and the dotted lines are the logarithmic extrapolations beyond the experimental sequencing depth.

Supplementary Table 11: Sequencing reads identified in the Blank samples. Enriched negative control blank libraries were sequenced on separate MiSeq 2 x 250 runs. After de-multiplexing, we pulled the reads that were associated with various index combinations used alongside the Blank Negative control throughout library preparation within the same trials and sets.

Sample	Samples processed alongside the blank library	Number of paired reads sequenced on run with Blank	Percentage of Blank
Blank Trial 1	C0002	1575	0.92
	C0018	0	0.00
	C0050	435	0.26
	C0060	379	0.22
	Pool1	3064	1.80
	Pool2	110959	65.05
	Pool3	36390	21.33
	Additional barcodes	2487	1.46
	Blank	15276	8.96
Blank Trial 2	C0002	6611	15.02
	C0018	11763	26.72
	C0050	5194	11.80
	C0060	4491	10.20
	Pool1	1178	2.68
	Pool2	4800	10.90
	Pool3	5862	13.31
	Additional barcodes	3044	6.91
	Blank	1083	2.46
Blank Set 1	1 - 1	456	17.61
	1 - 2	94	3.63
	1 - 3	174	6.72
	1 - 4	101	3.90
	1 – 5	316	12.20
	1 - 6	82	3.17
	1 - 7	683	26.37
	1 - 8	173	6.68
	1 - 9	35	1.35
	Negative Control - Blank	28	1.08
	C0002 – 1 - 1	120	4.63
	C0002 – 1 - 2	37	1.43

	C0002 – 1 - 3	291	11.24
Blank Set 2	2 - 1	367	9.65
	2 - 2	22	0.58
	2 - 3	44	1.16
	2 - 4	119	3.13
	2 - 5	40	1.05
	2 - 6	0	0.00
	2 - 7	39	1.03
	2 - 8	271	7.12
	2 - 9	137	3.60
	Negative Control - Blank	530	13.93
	C0002 – 2 -1	207	5.44
	C0002 – 2 - 2	34	0.89
	C0002 – 2 - 3	1994	52.42
Blank Set 3	3 - 1	224	3.76
	3 - 2	286	4.80
	3 - 3	71	1.19
	3 - 4	1653	27.73
	3 - 5	282	4.73
	3 - 6	23	0.39
	3 - 7	42	0.70
	3 - 8	128	2.15
	3 - 9	1198	20.09
	Negative Control - Blank	0	0.00
	C0002 – 3 -1	161	2.70
	C0002 – 3 - 2	817	13.70
	C0002 – 3 - 3	1077	18.06

Supplementary Table 12: Negative control enrichment with Blank samples. Enriched reds were divided among index combinations used during the respective Phase, Trial or Set (Supplementary Table 7). The reads belonging to each Negative Control – Blank library were trimmed and duplicates were removed then mapped to CARD through rgibwt. The number of genes with 1, at least 10 and at least 100 reads as well as genes with probes mapping, with average read mapping quality >=11 and gene length coverage with reads >=10% are shown. In Phase 2 Set 1, raw sequencing reads were used for analysis, in Set 2, deduplication was omitted, and for Set 3, there were no reads associated with the Blank indexes after sequencing.

Sample	Paired reads	Paired reads after trimming and de-duplication	Percent of reads mapping to CARD	Total genes with reads	Genes with 10 or more reads	Genes with 100 or more reads	Genes with at least 10 reads, >10% read coverage, MQ >=11 and probes
Blank Phase 1 Trial 1	15276	2716	80.34	153	82	9	10: *cpxA, mefA, arlS, mdtO, mdtE, mdtN, acrD, armA, AAC(3)-IV, APH(7'')-Ia,*
Blank Phase 1 Trial 2	1083	341	97.21	106	9	1	0
Phase 2 Set 1	28	N/A	0	0	0	0	0
Phase 2 Set 2*	530	412	76.46	94	26	0	**19**: *APH(3'')-Ib, acrD, acrE, acrF, acrS, cpxA, dfrA17, emrK, emrY, eptA, evgS, mdtE, mdtF, mdtH, mdtO, mdtP, pmrF, tetQ, tolC*
Phase 2 Set 3	0	0	0	0	0	0	0

*Genes in Set 2 Blank found in enriched and shotgun libraries: *tetQ, acrF*. Genes found in blank and enriched: *acrD, acrE*, *acrS*, *cpxA*, *emrK, emrY, eptA, evgS, mdtE, mdtF, mdtH, mdtO, mdtP*, *pmrF*, *tolC*. Genes found in blank only: *APH(3'')-Ib, dfrA17,*

Supplementary Table 13: Genes identified through metagenomic analysis of enriched and shotgun samples. Combining raw read counts across all 27 enriched and 6 shotgun sample at the full number of genes with the breakdown of gene, class and mechanisms identified. Genes were filtered based on genes with at least 10 reads mapping, percent coverage greater than or equal to 10%, mapping

quality greater than or equal to 11 and probes mapping (only for the enriched samples). This table is split into 4 parts with each part corresponding to a group of samples (Set 1, Set 2, Set 3 and the Shotgun samples). The first 6 columns are the same in all four parts.

ARO	Name	Class of Resistance	Present in Enriched	Present in Shotgun	Baits	Set 1 - 3	Set 1 - 4	Set 1 - 7	Set 1 - 6	Set 1 - 9	Set 1 - 8	Set 1 - 5	Set 1 - 2	Set 1 - 1
3000190	*tetO*	tetracycline antibiotic	27	6	Yes	2240	2088	655	3095	1195	2459	2613	2472	2909
3000191	*tetQ*	tetracycline antibiotic	27	6	Yes	21747	21337	7489	30223	13830	27383	22368	25974	25651
3000196	*tet32*	tetracycline antibiotic	27	6	Yes	5306	4929	1610	7133	2788	6253	5760	5554	6339
3000567	*tet(40)*	tetracycline antibiotic	27	6	Yes	4375	3252	978	5835	1891	4454	4654	3774	4098
3002837	*lnuC*	lincosamide antibiotic	27	6	Yes	2403	2223	828	2740	1202	2523	2240	2590	2884
3002867	*dfrF*	diaminopyrimidine antibiotic	27	6	Yes	1093	1242	412	1185	485	1126	1232	1296	1770
3002999	*cblA-1*	cephalosporin	27	6	Yes	2531	2026	743	3297	1182	2927	2612	2258	2268
3002926	*vanRG*	glycopeptide antibiotic	25	6	Yes	16	15	0	39	0	20	24	10	22
3000194	*tetW*	tetracycline antibiotic	0	6	No	0	0	0	0	0	0	0	0	0
3000375	*ermB*	macrolide antibiotic; lincosamide antibiotic; streptogramin antibiotic	0	6	No	0	0	0	0	0	0	0	0	0
3000501	Nocardia rifampin resistant beta-subunit of RNA polymerase (*rpoB2*)	peptide antibiotic; rifamycin antibiotic	0	6	No	0	0	0	0	0	0	0	0	0
3002522	*novA*	aminocoumarin antibiotic	0	6	Yes	0	0	0	0	0	0	0	0	0
3002597	aac(6')-Ie-aph(2")-Ia	aminoglycoside antibiotic	0	6	No	0	0	0	0	0	0	0	0	0
3003318	Streptomyces rishiriensis *parY* mutant conferring resistance to aminocoumarin	aminocoumarin antibiotic	0	6	No	0	0	0	0	0	0	0	0	0

3003730	Bifidobacteria intrinsic *ileS* conferring resistance to mupirocin	mupirocin	0	6	No	0	0	0	0	0	0	0	0	0
3004454	*Campylobacter coli* chloramphenicol acetyltransferase	phenicol antibiotic	0	6	No	0	0	0	0	0	0	0	0	0
3002965	*vanWG*	glycopeptide antibiotic	26	4	Yes	50	25	0	41	24	74	48	52	57
3000535	*macB*	macrolide antibiotic	22	4	Yes	26	0	0	107	0	43	56	0	29
3002647	*aph(3')-IIIa*	aminoglycoside antibiotic	0	3	No	0	0	0	0	0	0	0	0	0
3000556	*tet44*	tetracycline antibiotic	27	2	Yes	82	111	28	90	27	111	101	91	144
3003056	*smeE*	macrolide antibiotic; fluoroquinolone antibiotic; tetracycline antibiotic; phenicol antibiotic	8	2	Yes	16	0	0	13	0	10	0	0	0
3002937	*vanSG*	glycopeptide antibiotic	0	2	Yes	0	0	0	0	0	0	0	0	0
3002983	*amrB*	aminoglycoside antibiotic	0	2	No	0	0	0	0	0	0	0	0	0
3003559	*cepA* beta-lactamase	cephalosporin	0	2	Yes	0	0	0	0	0	0	0	0	0
3004032	*tetA(46)*	tetracycline antibiotic	0	2	No	0	0	0	0	0	0	0	0	0
3004033	*tetB(46)*	tetracycline antibiotic	0	2	No	0	0	0	0	0	0	0	0	0
3004074	*muxB*	macrolide antibiotic; monobactam; tetracycline antibiotic; aminocoumarin antibiotic	0	2	No	0	0	0	0	0	0	0	0	0
3004144	*axyY*	macrolide antibiotic; fluoroquinolone antibiotic; aminoglycoside	0	2	No	0	0	0	0	0	0	0	0	0

		antibiotic; cephalosporin												
3000502	*acrF*	fluoroquinolone antibiotic; cephalosporin; cephamycin; penam	27	1	Yes	190	107	28	181	51	140	141	127	130
3000793	*mdtB*	aminocoumarin antibiotic	0	1	No	0	0	0	0	0	0	0	0	0
3000794	*mdtC*	aminocoumarin antibiotic	0	1	No	0	0	0	0	0	0	0	0	0
3003097	*cfxA6*	cephamycin	0	1	Yes	0	0	0	0	0	0	0	0	0
3000027	*emrA*	fluoroquinolone antibiotic	27	0	Yes	49	51	20	40	26	51	39	37	38
3000237	*tolC*	macrolide antibiotic; fluoroquinolone antibiotic; cephalosporin; glycylcycline; cephamycin; penam; tetracycline antibiotic; aminocoumarin antibiotic; rifamycin antibiotic; phenicol antibiotic; triclosan	27	0	Yes	39	36	28	57	26	102	53	53	94
3000491	*acrD*	aminoglycoside antibiotic	27	0	Yes	83	66	24	173	36	111	143	83	88
3000615	*mefA*	macrolide antibiotic	27	0	Yes	57	27	13	55	27	68	33	36	43
3000616	*mel*	macrolide antibiotic; streptogramin antibiotic	27	0	Yes	28	11	13	50	11	36	53	69	73
3000795	*mdtE*	macrolide antibiotic; fluoroquinolone antibiotic; penam	27	0	Yes	92	64	12	173	38	78	125	56	96

3000796	*mdtF*	macrolide antibiotic; fluoroquinolone antibiotic; penam	27	0	Yes	144	102	22	223	76	110	94	102	131
3000830	*cpxA*	aminoglycoside antibiotic; aminocoumarin antibiotic	27	0	Yes	93	40	12	97	49	56	66	54	49
3000833	*evgS*	macrolide antibiotic; fluoroquinolone antibiotic; penam; tetracycline antibiotic	27	0	Yes	46	55	11	49	28	42	27	18	19
3001216	*mdtH*	fluoroquinolone antibiotic	27	0	Yes	23	55	11	73	19	35	35	66	23
3001328	*Escherichia coli mdfA*	tetracycline antibiotic; benzalkonium chloride; rhodamine	27	0	Yes	44	28	11	22	17	42	37	19	32
3003549	*mdtO*	nucleoside antibiotic; acridine dye	27	0	Yes	75	91	20	104	36	93	112	79	118
3003550	*mdtP*	nucleoside antibiotic; acridine dye	27	0	Yes	73	44	34	118	53	83	74	74	57
3003576	*eptA*	peptide antibiotic	27	0	Yes	59	76	16	112	30	65	65	68	91
3003578	*pmrF*	peptide antibiotic	27	0	Yes	68	25	11	71	30	42	46	53	47
3000074	*emrB*	fluoroquinolone antibiotic	26	0	Yes	42	15	0	36	15	29	48	43	19
3000499	*acrE*	fluoroquinolone antibiotic; cephalosporin; cephamycin; penam	26	0	Yes	68	37	0	76	24	40	66	56	48
3000518	*crp*	macrolide antibiotic; fluoroquinolone antibiotic; penam	26	0	Yes	31	10	0	47	17	28	21	16	10

3000656	*acrS*	fluoroquinolone antibiotic; cephalosporin; glycylcycline; cephamycin; penam; tetracycline antibiotic; rifamycin antibiotic; phenicol antibiotic; triclosan	26	0	Yes	23	28	0	36	26	18	16	11	37
3002635	*aph(2'')-IIa*	aminoglycoside antibiotic	26	0	Yes	59	31	15	65	0	35	29	51	46
3003548	*mdtN*	nucleoside antibiotic; acridine dye	26	0	Yes	57	40	0	33	15	18	24	17	11
3000254	*emrY*	tetracycline antibiotic	25	0	Yes	40	17	0	25	18	26	14	38	37
3001329	*mdtG*	fosfomycin	25	0	Yes	24	38	0	36	0	12	30	60	12
3002986	*bacA*	peptide antibiotic	25	0	Yes	27	33	0	35	0	42	20	15	17
3000216	*acrB*	fluoroquinolone antibiotic; cephalosporin; glycylcycline; penam; tetracycline antibiotic; rifamycin antibiotic; phenicol antibiotic; triclosan	24	0	Yes	13	13	0	35	0	25	14	16	0
3002688	*catS*	phenicol antibiotic	24	0	Yes	0	13	0	14	0	25	24	14	21
3000195	*tetB(P)*	tetracycline antibiotic	23	0	Yes	15	0	0	15	0	0	19	19	15
3000300	*lsaA*	lincosamide antibiotic; streptogramin antibiotic; pleuromutilin antibiotic	22	0	Yes	22	0	0	0	14	17	11	12	13

3000676	*H-NS*	macrolide antibiotic; fluoroquinolone antibiotic; cephalosporin; cephamycin; penam; tetracycline antibiotic	22	0	Yes	0	0	0	18	0	11	19	18	0
3003070	*vanXD*	glycopeptide antibiotic	22	0	Yes	25	19	0	22	0	0	19	0	0
3000180	*tetA(P)*	tetracycline antibiotic	21	0	Yes	15	15	0	0	0	0	0	12	31
3000593	*ermQ*	macrolide antibiotic; lincosamide antibiotic; streptogramin antibiotic	21	0	Yes	0	0	0	10	0	12	0	23	15
3002626	ant(6)-Ia	aminoglycoside antibiotic	21	0	Yes	17	0	0	0	11	0	0	11	0
3003069	*vanXYG*	glycopeptide antibiotic	21	0	Yes	27	0	0	13	0	0	12	0	0
3003206	*lsaE*	lincosamide antibiotic; pleuromutilin antibiotic	21	0	Yes	13	0	0	15	0	16	0	0	31
3000206	*emrK*	tetracycline antibiotic	20	0	Yes	0	0	0	0	0	14	12	0	13
3003551	*emeA*	acridine dye	20	0	Yes	0	22	0	0	0	0	13	16	0
3002923	*vanRD*	glycopeptide antibiotic	19	0	Yes	0	0	0	0	0	0	13	0	13
3002944	*vanHD*	glycopeptide antibiotic	19	0	Yes	15	0	0	17	0	0	12	0	0
3000005	*vanD*	glycopeptide antibiotic	18	0	Yes	0	15	0	28	0	0	0	12	0
3000522	*ermG*	macrolide antibiotic; lincosamide antibiotic; streptogramin antibiotic	17	0	Yes	29	15	0	11	0	0	10	0	10

3000263	*marA*	fluoroquinolone antibiotic; monobactam; carbapenem; cephalosporin; glycylcycline; cephamycin; penam; tetracycline antibiotic; rifamycin antibiotic; phenicol antibiotic; triclosan; penem	14	0	Yes	0	0	0	0	0	0	0	10	10
3000832	*evgA*	macrolide antibiotic; fluoroquinolone antibiotic; penam; tetracycline antibiotic	14	0	Yes	0	15	0	0	0	0	0	0	0
3002972	*vanTG*	glycopeptide antibiotic	14	0	Yes	0	0	0	0	0	0	0	10	14
3002630	*ant(9)-Ia*	aminoglycoside antibiotic	12	0	Yes	0	0	0	0	0	0	0	15	0
3000508	*gadX*	macrolide antibiotic; fluoroquinolone antibiotic; penam	11	0	Yes	0	0	0	21	0	0	0	0	0
3002882	*lmrD*	lincosamide antibiotic	10	0	Yes	0	0	0	0	0	16	0	0	0
3002957	*vanYD*	glycopeptide antibiotic	10	0	Yes	0	0	0	0	0	0	0	10	14
3001214	*mdtM*	fluoroquinolone antibiotic; lincosamide antibiotic; nucleoside antibiotic; acridine dye; phenicol antibiotic	7	0	Yes	0	0	0	0	0	0	0	0	0
3002909	*vanG*	glycopeptide antibiotic	6	0	Yes	0	0	0	0	0	0	0	0	0

3003112	*lsaC*	lincosamide antibiotic; streptogramin antibiotic; pleuromutilin antibiotic	6	0	Yes	0	0	0	0	0	0	0	0	0
3002881	*lmrC*	lincosamide antibiotic	4	0	Yes	0	0	0	0	0	0	0	0	0
3000792	*mdtA*	aminocoumarin antibiotic	3	0	Yes	10	0	0	0	0	0	0	0	0
3000186	*tetM*	tetracycline antibiotic	2	0	Yes	0	0	0	0	0	0	0	0	0
3000801	*mexD*	macrolide antibiotic; fluoroquinolone antibiotic; aminoglycoside antibiotic; cephalosporin; penam; tetracycline antibiotic; aminocoumarin antibiotic; diaminopyrimidine antibiotic; phenicol antibiotic	2	0	Yes	0	0	0	0	0	0	0	0	0
3003052	*smeB*	aminoglycoside antibiotic; cephalosporin; cephamycin; penam	2	0	Yes	0	0	0	0	0	0	0	0	0
3002629	*ant(6)-Ib*	aminoglycoside antibiotic	1	0	Yes	0	0	0	0	0	0	0	0	0

**ARO**	**Name**	**Class of Resistance**	**Present in Enriched**	**Present in Shotgun**	**Baits**	**Set 2 - 9**	**Set 2 - 3**	**Set 2 - 2**	**Set 2 - 6**	**Set 2 - 5**	**Set 2 - 1**	**Set 2 - 4**	**Set 2 - 8**	**Set 2 - 7**
3000190	*tetO*	tetracycline antibiotic	27	6	Yes	3478	4231	4417	6684	6400	5670	5324	6567	4717
3000191	*tetQ*	tetracycline antibiotic	27	6	Yes	22674	32260	29099	46576	50381	46810	31557	36461	28754
3000196	*tet32*	tetracycline antibiotic	27	6	Yes	6678	8515	8709	12551	12546	11884	9807	11034	9021
3000567	*tet(40)*	tetracycline antibiotic	27	6	Yes	5956	7154	6153	10967	9134	7174	7321	9325	7143
3002837	*lnuC*	lincosamide antibiotic	27	6	Yes	2443	3407	3560	4857	5135	5372	3895	4083	3376
3002867	*dfrF*	diaminopyrimidine antibiotic	27	6	Yes	1286	1855	2000	2435	2620	3186	2469	2272	1916

3002999	*cblA-1*	cephalosporin	27	6	Yes	2970	3701	3263	5479	4788	4264	3771	4510	3451
3002926	*vanRG*	glycopeptide antibiotic	25	6	Yes	52	44	17	74	63	56	41	78	43
3000194	*tetW*	tetracycline antibiotic	0	6	No	0	0	0	0	0	0	0	0	0
3000375	*ermB*	macrolide antibiotic; lincosamide antibiotic; streptogramin antibiotic	0	6	No	0	0	0	0	0	0	0	0	0
3000501	Nocardia rifampin resistant beta-subunit of RNA polymerase (*rpoB2*)	peptide antibiotic; rifamycin antibiotic	0	6	No	0	0	0	0	0	0	0	0	0
3002522	*novA*	aminocoumarin antibiotic	0	6	Yes	0	0	0	0	0	0	0	0	0
3002597	aac(6')-Ie-aph(2")-Ia	aminoglycoside antibiotic	0	6	No	0	0	0	0	0	0	0	0	0
3003318	Streptomyces rishiriensis *parY* mutant conferring resistance to aminocoumarin	aminocoumarin antibiotic	0	6	No	0	0	0	0	0	0	0	0	0
3003730	Bifidobacteria intrinsic *ileS* conferring resistance to mupirocin	mupirocin	0	6	No	0	0	0	0	0	0	0	0	0
3004454	*Campylobacter coli* chloramphenicol acetyltransferase	phenicol antibiotic	0	6	No	0	0	0	0	0	0	0	0	0
3002965	*vanWG*	glycopeptide antibiotic	26	4	Yes	86	121	91	193	184	120	109	178	135
3000535	*macB*	macrolide antibiotic	22	4	Yes	106	84	62	167	95	66	65	96	75
3002647	*aph(3')-IIIa*	aminoglycoside antibiotic	0	3	No	0	0	0	0	0	0	0	0	0
3000556	*tet44*	tetracycline antibiotic	27	2	Yes	115	172	200	252	283	279	271	277	210
3003056	*smeE*	macrolide antibiotic;	8	2	Yes	0	18	0	22	15	0	0	16	0

		fluoroquinolone antibiotic; tetracycline antibiotic; phenicol antibiotic												
3002937	*vanSG*	glycopeptide antibiotic	0	2	Yes	0	0	0	0	0	0	0	0	0
3002983	*amrB*	aminoglycoside antibiotic	0	2	No	0	0	0	0	0	0	0	0	0
3003559	*cepA* beta-lactamase	cephalosporin	0	2	Yes	0	0	0	0	0	0	0	0	0
3004032	*tetA(46)*	tetracycline antibiotic	0	2	No	0	0	0	0	0	0	0	0	0
3004033	*tetB(46)*	tetracycline antibiotic	0	2	No	0	0	0	0	0	0	0	0	0
3004074	*muxB*	macrolide antibiotic; monobactam; tetracycline antibiotic; aminocoumarin antibiotic	0	2	No	0	0	0	0	0	0	0	0	0
3004144	*axyY*	macrolide antibiotic; fluoroquinolone antibiotic; aminoglycoside antibiotic; cephalosporin	0	2	No	0	0	0	0	0	0	0	0	0
3000502	*acrF*	fluoroquinolone antibiotic; cephalosporin; cephamycin; penam	27	1	Yes	229	310	209	466	377	290	218	407	221
3000793	*mdtB*	aminocoumarin antibiotic	0	1	No	0	0	0	0	0	0	0	0	0
3000794	*mdtC*	aminocoumarin antibiotic	0	1	No	0	0	0	0	0	0	0	0	0
3003097	*cfxA6*	cephamycin	0	1	Yes	0	0	0	0	0	0	0	0	0
3000027	*emrA*	fluoroquinolone antibiotic	27	0	Yes	94	119	98	139	123	91	98	149	121
3000237	*tolC*	macrolide antibiotic; fluoroquinolone antibiotic; cephalosporin; glycylcycline; cephamycin; penam;	27	0	Yes	75	100	102	186	142	113	127	158	69

		tetracycline antibiotic; aminocoumarin antibiotic; rifamycin antibiotic; phenicol antibiotic; triclosan												
3000491	*acrD*	aminoglycoside antibiotic	27	0	Yes	186	217	170	398	281	111	180	337	192
3000615	*mefA*	macrolide antibiotic	27	0	Yes	82	75	92	134	168	100	107	114	118
3000616	*mel*	macrolide antibiotic; streptogramin antibiotic	27	0	Yes	93	60	80	163	166	168	139	162	162
3000795	*mdtE*	macrolide antibiotic; fluoroquinolone antibiotic; penam	27	0	Yes	127	176	127	293	271	148	199	228	134
3000796	*mdtF*	macrolide antibiotic; fluoroquinolone antibiotic; penam	27	0	Yes	208	267	163	455	292	190	254	350	181
3000830	*cpxA*	aminoglycoside antibiotic; aminocoumarin antibiotic	27	0	Yes	135	147	112	290	215	132	96	215	140
3000833	*evgS*	macrolide antibiotic; fluoroquinolone antibiotic; penam; tetracycline antibiotic	27	0	Yes	43	62	88	120	130	126	76	116	53
3001216	*mdtH*	fluoroquinolone antibiotic	27	0	Yes	52	69	74	137	86	63	62	67	43
3001328	*Escherichia coli mdfA*	tetracycline antibiotic; benzalkonium chloride; rhodamine	27	0	Yes	49	98	51	100	44	77	60	105	66
3003549	*mdtO*	nucleoside antibiotic; acridine dye	27	0	Yes	129	197	164	290	262	119	145	241	162

3003550	*mdtP*	nucleoside antibiotic; acridine dye	27	0	Yes	135	140	116	252	266	136	102	192	154
3003576	*eptA*	peptide antibiotic	27	0	Yes	121	121	109	234	171	139	111	208	91
3003578	*pmrF*	peptide antibiotic	27	0	Yes	89	128	82	182	151	77	89	151	95
3000074	*emrB*	fluoroquinolone antibiotic	26	0	Yes	86	80	49	151	88	76	50	127	70
3000499	*acrE*	fluoroquinolone antibiotic; cephalosporin; cephamycin; penam	26	0	Yes	90	107	76	178	102	151	82	149	82
3000518	*crp*	macrolide antibiotic; fluoroquinolone antibiotic; penam	26	0	Yes	36	80	35	90	48	54	47	54	29
3000656	*acrS*	fluoroquinolone antibiotic; cephalosporin; glycylcycline; cephamycin; penam; tetracycline antibiotic; rifamycin antibiotic; phenicol antibiotic; triclosan	26	0	Yes	67	52	52	101	83	62	44	93	78
3002635	*aph(2'')-IIa*	aminoglycoside antibiotic	26	0	Yes	50	43	69	84	102	97	138	90	93
3003548	*mdtN*	nucleoside antibiotic; acridine dye	26	0	Yes	43	76	47	105	85	41	57	81	25
3000254	*emrY*	tetracycline antibiotic	25	0	Yes	28	11	23	71	31	49	33	61	44
3001329	*mdtG*	fosfomycin	25	0	Yes	42	47	44	97	94	28	74	113	44
3002986	*bacA*	peptide antibiotic	25	0	Yes	40	48	30	70	65	15	44	51	42
3000216	*acrB*	fluoroquinolone antibiotic; cephalosporin; glycylcycline; penam; tetracycline antibiotic; rifamycin	24	0	Yes	45	40	28	61	36	17	22	34	22

3002688	*catS*	antibiotic; phenicol antibiotic; triclosan phenicol antibiotic	24	0	Yes	22	39	42	63	57	69	39	40	36
3000195	*tetB(P)*	tetracycline antibiotic	23	0	Yes	25	27	31	30	48	55	40	24	28
3000300	*lsaA*	lincosamide antibiotic; streptogramin antibiotic; pleuromutilin antibiotic	22	0	Yes	17	15	28	28	24	30	37	23	35
3000676	*H-NS*	macrolide antibiotic; fluoroquinolone antibiotic; cephalosporin; cephamycin; penam; tetracycline antibiotic	22	0	Yes	50	37	20	56	56	37	27	62	41
3003070	*vanXD*	glycopeptide antibiotic	22	0	Yes	12	27	16	32	26	12	24	39	22
3000180	*tetA(P)*	tetracycline antibiotic	21	0	Yes	19	17	31	43	21	30	46	52	28
3000593	*ermQ*	macrolide antibiotic; lincosamide antibiotic; streptogramin antibiotic	21	0	Yes	11	13	11	33	34	29	25	16	18
3002626	ant(6)-Ia	aminoglycoside antibiotic	21	0	Yes	14	17	14	29	25	18	26	27	28
3003069	*vanXYG*	glycopeptide antibiotic	21	0	Yes	26	19	16	40	50	29	28	37	20
3003206	*lsaE*	lincosamide antibiotic; pleuromutilin antibiotic	21	0	Yes	20	30	25	34	27	53	28	42	30
3000206	*emrK*	tetracycline antibiotic	20	0	Yes	29	29	24	39	25	22	35	34	32
3003551	*emeA*	acridine dye	20	0	Yes	22	28	26	31	16	29	34	39	44
3002923	*vanRD*	glycopeptide antibiotic	19	0	Yes	13	19	12	42	25	28	14	36	15
3002944	*vanHD*	glycopeptide antibiotic	19	0	Yes	0	18	37	37	30	17	32	26	23
3000005	*vanD*	glycopeptide antibiotic	18	0	Yes	0	26	16	29	28	44	17	19	26

3000522	*ermG*	macrolide antibiotic; lincosamide antibiotic; streptogramin antibiotic	17	0	Yes	0	22	23	16	15	27	27	21	19
3000263	*marA*	fluoroquinolone antibiotic; monobactam; carbapenem; cephalosporin; glycylcycline; cephamycin; penam; tetracycline antibiotic; rifamycin antibiotic; phenicol antibiotic; triclosan; penem	14	0	Yes	14	17	10	28	16	20	20	22	0
3000832	*evgA*	macrolide antibiotic; fluoroquinolone antibiotic; penam; tetracycline antibiotic	14	0	Yes	10	0	12	23	15	12	13	26	17
3002972	*vanTG*	glycopeptide antibiotic	14	0	Yes	12	13	0	24	26	12	14	13	19
3002630	*ant(9)-Ia*	aminoglycoside antibiotic	12	0	Yes	0	11	0	10	0	13	22	16	0
3000508	*gadX*	macrolide antibiotic; fluoroquinolone antibiotic; penam	11	0	Yes	16	0	12	16	17	0	19	0	11
3002882	*lmrD*	lincosamide antibiotic	10	0	Yes	0	0	28	0	0	13	14	0	12
3002957	*vanYD*	glycopeptide antibiotic	10	0	Yes	0	0	12	10	10	0	0	11	0
3001214	*mdtM*	fluoroquinolone antibiotic; lincosamide antibiotic; nucleoside antibiotic; acridine dye;	7	0	Yes	10	0	0	15	0	16	11	16	0

		phenicol antibiotic												
3002909	*vanG*	glycopeptide antibiotic	6	0	Yes	0	0	0	0	15	14	16	12	0
3003112	*lsaC*	lincosamide antibiotic; streptogramin antibiotic; pleuromutilin antibiotic	6	0	Yes	0	0	0	17	12	0	18	15	14
3002881	*lmrC*	lincosamide antibiotic	4	0	Yes	0	0	0	0	0	0	0	12	17
3000792	*mdtA*	aminocoumarin antibiotic	3	0	Yes	0	0	0	0	0	19	0	0	0
3000186	*tetM*	tetracycline antibiotic	2	0	Yes	0	0	0	0	0	0	0	11	0
3000801	*mexD*	macrolide antibiotic; fluoroquinolone antibiotic; aminoglycoside antibiotic; cephalosporin; penam; tetracycline antibiotic; aminocoumarin antibiotic; diaminopyrimidine antibiotic; phenicol antibiotic	2	0	Yes	0	0	0	16	0	0	0	0	0
3003052	*smeB*	aminoglycoside antibiotic; cephalosporin; cephamycin; penam	2	0	Yes	0	0	0	0	0	0	0	0	0
3002629	*ant(6)-Ib*	aminoglycoside antibiotic	1	0	Yes	0	0	0	0	0	0	0	0	0

**ARO**	**Name**	**Class of Resistance**	**Present in Enriched**	**Present in Shotgun**	**Baits**	**Set 3 - 9**	**Set 3 - 6**	**Set 3 - 8**	**Set 3 - 7**	**Set 3 - 5**	**Set 3 - 3**	**Set 3 - 2**	**Set 3 - 4**	**Set 3 - 1**
3000190	*tetO*	tetracycline antibiotic	27	6	Yes	4389	3143	4035	4083	3662	3459	5115	3742	4278
3000191	*tetQ*	tetracycline antibiotic	27	6	Yes	31961	25807	27902	30217	30537	31375	57377	35805	38948
3000196	*tet32*	tetracycline antibiotic	27	6	Yes	8770	7045	8207	8497	8055	7484	12627	9006	9549
3000567	*tet(40)*	tetracycline antibiotic	27	6	Yes	7844	5526	7038	6490	6893	5856	7884	5888	5971

3002837	*lnuC*	lincosamide antibiotic	27	6	Yes	3591	2944	3308	3659	3351	3360	6483	4322	4901
3002867	*dfrF*	diaminopyrimidine antibiotic	27	6	Yes	1624	1429	1733	2133	1746	1579	3276	2464	2945
3002999	*cblA-1*	cephalosporin	27	6	Yes	4441	3146	4007	4244	3884	3509	5435	3914	3948
3002926	*vanRG*	glycopeptide antibiotic	25	6	Yes	49	50	29	19	29	45	21	18	21
3000194	*tetW*	tetracycline antibiotic	0	6	No	0	0	0	0	0	0	0	0	0
3000375	*ermB*	macrolide antibiotic; lincosamide antibiotic; streptogramin antibiotic	0	6	No	0	0	0	0	0	0	0	0	0
3000501	Nocardia rifampin resistant beta-subunit of RNA polymerase (*rpoB2*)	peptide antibiotic; rifamycin antibiotic	0	6	No	0	0	0	0	0	0	0	0	0
3002522	*novA*	aminocoumarin antibiotic	0	6	Yes	0	0	0	0	0	0	0	0	0
3002597	aac(6')-Ie-aph(2")-Ia	aminoglycoside antibiotic	0	6	No	0	0	0	0	0	0	0	0	0
3003318	Streptomyces rishiriensis *parY* mutant conferring resistance to aminocoumarin	aminocoumarin antibiotic	0	6	No	0	0	0	0	0	0	0	0	0
3003730	Bifidobacteria intrinsic *ileS* conferring resistance to mupirocin	mupirocin	0	6	No	0	0	0	0	0	0	0	0	0
3004454	*Campylobacter coli* chloramphenicol acetyltransferase	phenicol antibiotic	0	6	No	0	0	0	0	0	0	0	0	0
3002965	*vanWG*	glycopeptide antibiotic	26	4	Yes	109	78	107	89	76	80	94	70	107
3000535	*macB*	macrolide antibiotic	22	4	Yes	92	71	82	77	63	87	0	51	51
3002647	*aph(3')-IIIa*	aminoglycoside antibiotic	0	3	No	0	0	0	0	0	0	0	0	0

3000556	*tet44*	tetracycline antibiotic	27	2	Yes	145	110	130	117	119	112	216	170	211
3003056	*smeE*	macrolide antibiotic; fluoroquinolone antibiotic; tetracycline antibiotic; phenicol antibiotic	8	2	Yes	0	0	0	0	20	0	0	0	0
3002937	*vanSG*	glycopeptide antibiotic	0	2	Yes	0	0	0	0	0	0	0	0	0
3002983	*amrB*	aminoglycoside antibiotic	0	2	No	0	0	0	0	0	0	0	0	0
3003559	*cepA* beta-lactamase	cephalosporin	0	2	Yes	0	0	0	0	0	0	0	0	0
3004032	*tetA(46)*	tetracycline antibiotic	0	2	No	0	0	0	0	0	0	0	0	0
3004033	*tetB(46)*	tetracycline antibiotic	0	2	No	0	0	0	0	0	0	0	0	0
3004074	*muxB*	macrolide antibiotic; monobactam; tetracycline antibiotic; aminocoumarin antibiotic	0	2	No	0	0	0	0	0	0	0	0	0
3004144	*axyY*	macrolide antibiotic; fluoroquinolone antibiotic; aminoglycoside antibiotic; cephalosporin	0	2	No	0	0	0	0	0	0	0	0	0
3000502	*acrF*	fluoroquinolone antibiotic; cephalosporin; cephamycin; penam	27	1	Yes	219	188	188	171	199	166	192	154	155
3000793	*mdtB*	aminocoumarin antibiotic	0	1	No	0	0	0	0	0	0	0	0	0
3000794	*mdtC*	aminocoumarin antibiotic	0	1	No	0	0	0	0	0	0	0	0	0
3003097	*cfxA6*	cephamycin	0	1	Yes	0	0	0	0	0	0	0	0	0
3000027	*emrA*	fluoroquinolone antibiotic	27	0	Yes	72	67	70	80	80	53	87	55	94
3000237	*tolC*	macrolide antibiotic; fluoroquinolone antibiotic;	27	0	Yes	81	59	60	90	109	64	74	75	83

		cephalosporin; glycylcycline; cephamycin; penam; tetracycline antibiotic; aminocoumarin antibiotic; rifamycin antibiotic; phenicol antibiotic; triclosan												
3000491	*acrD*	aminoglycoside antibiotic	27	0	Yes	145	161	182	152	254	150	165	134	126
3000615	*mefA*	macrolide antibiotic	27	0	Yes	79	57	73	56	54	56	70	84	58
3000616	*mel*	macrolide antibiotic; streptogramin antibiotic	27	0	Yes	90	61	75	98	63	63	165	119	114
3000795	*mdtE*	macrolide antibiotic; fluoroquinolone antibiotic; penam	27	0	Yes	139	64	118	83	127	110	114	110	93
3000796	*mdtF*	macrolide antibiotic; fluoroquinolone antibiotic; penam	27	0	Yes	247	155	156	149	172	113	159	159	134
3000830	*cpxA*	aminoglycoside antibiotic; aminocoumarin antibiotic	27	0	Yes	142	101	91	94	133	131	108	90	85
3000833	*evgS*	macrolide antibiotic; fluoroquinolone antibiotic; penam; tetracycline antibiotic	27	0	Yes	36	47	47	59	64	28	66	68	61
3001216	*mdtH*	fluoroquinolone antibiotic	27	0	Yes	72	31	38	39	34	37	56	47	45
3001328	*Escherichia coli mdfA*	tetracycline antibiotic; benzalkonium chloride; rhodamine	27	0	Yes	40	47	46	54	48	42	46	43	50

3003549	*mdtO*	nucleoside antibiotic; acridine dye	27	0	Yes	124	88	124	107	132	126	127	101	104
3003550	*mdtP*	nucleoside antibiotic; acridine dye	27	0	Yes	138	103	154	110	128	115	134	96	71
3003576	*eptA*	peptide antibiotic	27	0	Yes	125	87	75	76	113	79	84	107	107
3003578	*pmrF*	peptide antibiotic	27	0	Yes	96	64	67	75	81	47	87	56	48
3000074	*emrB*	fluoroquinolone antibiotic	26	0	Yes	37	53	44	76	55	63	65	47	62
3000499	*acrE*	fluoroquinolone antibiotic; cephalosporin; cephamycin; penam	26	0	Yes	91	75	88	66	78	43	73	80	65
3000518	*crp*	macrolide antibiotic; fluoroquinolone antibiotic; penam	26	0	Yes	44	17	45	27	23	26	39	23	16
3000656	*acrS*	fluoroquinolone antibiotic; cephalosporin; glycylcycline; cephamycin; penam; tetracycline antibiotic; rifamycin antibiotic; phenicol antibiotic; triclosan	26	0	Yes	33	32	54	29	32	18	68	38	40
3002635	*aph(2'')-IIa*	aminoglycoside antibiotic	26	0	Yes	71	51	57	43	39	52	98	77	63
3003548	*mdtN*	nucleoside antibiotic; acridine dye	26	0	Yes	26	36	39	23	37	34	34	22	33
3000254	*emrY*	tetracycline antibiotic	25	0	Yes	33	0	30	20	23	18	32	55	29
3001329	*mdtG*	fosfomycin	25	0	Yes	31	44	32	45	45	38	40	15	27
3002986	*bacA*	peptide antibiotic	25	0	Yes	42	49	29	41	36	15	20	28	33
3000216	*acrB*	fluoroquinolone antibiotic; cephalosporin; glycylcycline; penam;	24	0	Yes	28	15	34	17	29	23	24	26	10

		tetracycline antibiotic; rifamycin antibiotic; phenicol antibiotic; triclosan												
3002688	*catS*	phenicol antibiotic	24	0	Yes	36	15	18	22	25	28	45	33	44
3000195	*tetB(P)*	tetracycline antibiotic	23	0	Yes	23	17	23	25	29	30	16	20	39
3000300	*lsaA*	lincosamide antibiotic; streptogramin antibiotic; pleuromutilin antibiotic	22	0	Yes	0	0	18	11	18	10	17	15	25
3000676	*H-NS*	macrolide antibiotic; fluoroquinolone antibiotic; cephalosporin; cephamycin; penam; tetracycline antibiotic	22	0	Yes	41	31	19	17	30	37	35	30	27
3003070	*vanXD*	glycopeptide antibiotic	22	0	Yes	16	13	12	11	18	19	54	21	25
3000180	*tetA(P)*	tetracycline antibiotic	21	0	Yes	25	14	28	31	33	0	36	45	48
3000593	*ermQ*	macrolide antibiotic; lincosamide antibiotic; streptogramin antibiotic	21	0	Yes	0	15	15	21	10	13	28	15	26
3002626	ant(6)-Ia	aminoglycoside antibiotic	21	0	Yes	19	26	14	15	13	15	23	19	12
3003069	*vanXYG*	glycopeptide antibiotic	21	0	Yes	23	15	25	10	24	20	11	13	20
3003206	*lsaE*	lincosamide antibiotic; pleuromutilin antibiotic	21	0	Yes	0	21	13	29	26	16	46	17	22
3000206	*emrK*	tetracycline antibiotic	20	0	Yes	15	13	10	0	15	16	12	20	25
3003551	*emeA*	acridine dye	20	0	Yes	16	29	32	22	0	13	20	20	35
3002923	*vanRD*	glycopeptide antibiotic	19	0	Yes	20	16	0	11	14	13	18	11	23

3002944	*vanHD*	glycopeptide antibiotic	19	0	Yes	15	22	0	37	14	19	32	11	32
3000005	*vanD*	glycopeptide antibiotic	18	0	Yes	14	0	19	20	19	0	15	13	14
3000522	*ermG*	macrolide antibiotic; lincosamide antibiotic; streptogramin antibiotic	17	0	Yes	0	0	18	0	0	13	31	10	0
3000263	*marA*	fluoroquinolone antibiotic; monobactam; carbapenem; cephalosporin; glycylcycline; cephamycin; penam; tetracycline antibiotic; rifamycin antibiotic; phenicol antibiotic; triclosan; penem	14	0	Yes	17	0	0	0	0	0	14	12	12
3000832	*evgA*	macrolide antibiotic; fluoroquinolone antibiotic; penam; tetracycline antibiotic	14	0	Yes	0	11	0	12	0	13	23	0	16
3002972	*vanTG*	glycopeptide antibiotic	14	0	Yes	0	0	12	0	0	12	0	20	11
3002630	*ant(9)-Ia*	aminoglycoside antibiotic	12	0	Yes	0	11	16	0	11	0	34	26	11
3000508	*gadX*	macrolide antibiotic; fluoroquinolone antibiotic; penam	11	0	Yes	0	0	0	13	13	0	15	12	0
3002882	*lmrD*	lincosamide antibiotic	10	0	Yes	19	0	0	0	0	10	18	14	12
3002957	*vanYD*	glycopeptide antibiotic	10	0	Yes	0	19	0	0	12	0	12	0	13
3001214	*mdtM*	fluoroquinolone antibiotic; lincosamide antibiotic;	7	0	Yes	0	0	10	0	10	0	0	0	0

		nucleoside antibiotic; acridine dye; phenicol antibiotic												
3002909	*vanG*	glycopeptide antibiotic	6	0	Yes	12	0	0	0	0	0	14	0	0
3003112	*lsaC*	lincosamide antibiotic; streptogramin antibiotic; pleuromutilin antibiotic	6	0	Yes	0	0	12	0	0	0	0	0	0
3002881	*lmrC*	lincosamide antibiotic	4	0	Yes	11	0	0	14	0	0	0	0	0
3000792	*mdtA*	aminocoumarin antibiotic	3	0	Yes	0	0	0	0	0	0	0	10	0
3000186	*tetM*	tetracycline antibiotic	2	0	Yes	0	0	0	0	0	0	0	13	0
3000801	*mexD*	macrolide antibiotic; fluoroquinolone antibiotic; aminoglycoside antibiotic; cephalosporin; penam; tetracycline antibiotic; aminocoumarin antibiotic; diaminopyrimidine antibiotic; phenicol antibiotic	2	0	Yes	0	0	0	0	17	0	0	0	0
3003052	*smeB*	aminoglycoside antibiotic; cephalosporin; cephamycin; penam	2	0	Yes	0	32	0	0	0	38	0	0	0
3002629	*ant(6)-Ib*	aminoglycoside antibiotic	1	0	Yes	0	0	0	0	0	0	10	0	0

**ARO**	**Name**	**Class of Resistance**	**Present in Enriched**	**Present in Shotgun**	**Baits**	**Set 1 - 6 Shotgun**	**Set 1 - 7 Shotgun**	**Set 2 - 9 Shotgun**	**Set 2 - 5 Shotgun**	**Set 3 - 5 Shotgun**	**Set 3 - 1 Shotgun**
3000190	*tetO*	tetracycline antibiotic	27	6	Yes	127	146	296	281	179	211
3000191	*tetQ*	tetracycline antibiotic	27	6	Yes	654	774	1568	1314	790	1150
3000196	*tet32*	tetracycline antibiotic	27	6	Yes	116	151	238	221	133	227

3000567	*tet(40)*	tetracycline antibiotic	27	6	Yes	44	59	96	90	66	72
3002837	*lnuC*	lincosamide antibiotic	27	6	Yes	94	114	208	174	84	152
3002867	*dfrF*	diaminopyrimidine antibiotic	27	6	Yes	32	32	86	50	38	48
3002999	*cblA-1*	cephalosporin	27	6	Yes	46	50	60	66	44	76
3002926	*vanRG*	glycopeptide antibiotic	25	6	Yes	10	22	30	28	16	24
3000194	*tetW*	tetracycline antibiotic	0	6	No	546	635	1108	836	649	862
3000375	*ermB*	macrolide antibiotic; lincosamide antibiotic; streptogramin antibiotic	0	6	No	36	34	74	70	34	46
3000501	Nocardia rifampin resistant beta-subunit of RNA polymerase (*rpoB2*)	peptide antibiotic; rifamycin antibiotic	0	6	No	86	120	136	94	96	108
3002522	*novA*	aminocoumarin antibiotic	0	6	Yes	12	14	14	24	22	16
3002597	aac(6')-Ie-aph(2")-Ia	aminoglycoside antibiotic	0	6	No	30	44	80	78	46	56
3003318	Streptomyces rishiriensis *parY* mutant conferring resistance to aminocoumarin	aminocoumarin antibiotic	0	6	No	96	108	178	148	110	124
3003730	Bifidobacteria intrinsic *ileS* conferring resistance to mupirocin	mupirocin	0	6	No	50	74	98	68	60	82
3004454	*Campylobacter coli* chloramphenicol acetyltransferase	phenicol antibiotic	0	6	No	14	16	22	26	10	22
3002965	*vanWG*	glycopeptide antibiotic	26	4	Yes	14	0	28	24	14	0
3000535	*macB*	macrolide antibiotic	22	4	Yes	0	12	16	28	0	18
3002647	*aph(3')-IIIa*	aminoglycoside antibiotic	0	3	No	0	12	0	10	0	10
3000556	*tet44*	tetracycline antibiotic	27	2	Yes	0	0	10	12	0	0

3003056	*smeE*	macrolide antibiotic; fluoroquinolone antibiotic; tetracycline antibiotic; phenicol antibiotic	8	2	Yes	0	12	12	0	0	0
3002937	*vanSG*	glycopeptide antibiotic	0	2	Yes	0	10	16	0	0	0
3002983	*amrB*	aminoglycoside antibiotic	0	2	No	0	0	0	0	10	10
3003559	*cepA* beta-lactamase	cephalosporin	0	2	Yes	0	0	12	0	10	0
3004032	*tetA(46)*	tetracycline antibiotic	0	2	No	0	0	10	0	0	16
3004033	*tetB(46)*	tetracycline antibiotic	0	2	No	0	0	14	10	0	0
3004074	*muxB*	macrolide antibiotic; monobactam; tetracycline antibiotic; aminocoumarin antibiotic	0	2	No	0	0	0	15	0	18
3004144	*axyY*	macrolide antibiotic; fluoroquinolone antibiotic; aminoglycoside antibiotic; cephalosporin	0	2	No	16	0	26	0	0	0
3000502	*acrF*	fluoroquinolone antibiotic; cephalosporin; cephamycin; penam	27	1	Yes	0	0	13	0	0	0
3000793	*mdtB*	aminocoumarin antibiotic	0	1	No	0	0	0	15	0	0
3000794	*mdtC*	aminocoumarin antibiotic	0	1	No	0	0	10	0	0	0
3003097	*cfxA6*	cephamycin	0	1	Yes	0	0	0	0	0	43
3000027	*emrA*	fluoroquinolone antibiotic	27	0	Yes	0	0	0	0	0	0

3000237	*tolC*	macrolide antibiotic; fluoroquinolone antibiotic; cephalosporin; glycylcycline; cephamycin; penam; tetracycline antibiotic; aminocoumarin antibiotic; rifamycin antibiotic; phenicol antibiotic; triclosan	27	0	Yes	0	0	0	0	0	0
3000491	*acrD*	aminoglycoside antibiotic	27	0	Yes	0	0	0	0	0	0
3000615	*mefA*	macrolide antibiotic	27	0	Yes	0	0	0	0	0	0
3000616	*mel*	macrolide antibiotic; streptogramin antibiotic	27	0	Yes	0	0	0	0	0	0
3000795	*mdtE*	macrolide antibiotic; fluoroquinolone antibiotic; penam	27	0	Yes	0	0	0	0	0	0
3000796	*mdtF*	macrolide antibiotic; fluoroquinolone antibiotic; penam	27	0	Yes	0	0	0	0	0	0
3000830	*cpxA*	aminoglycoside antibiotic; aminocoumarin antibiotic	27	0	Yes	0	0	0	0	0	0
3000833	*evgS*	macrolide antibiotic; fluoroquinolone antibiotic; penam; tetracycline antibiotic	27	0	Yes	0	0	0	0	0	0
3001216	*mdtH*	fluoroquinolone antibiotic	27	0	Yes	0	0	0	0	0	0
3001328	*Escherichia coli mdfA*	tetracycline antibiotic; benzalkonium chloride; rhodamine	27	0	Yes	0	0	0	0	0	0
3003549	*mdtO*	nucleoside antibiotic; acridine dye	27	0	Yes	0	0	0	0	0	0
3003550	*mdtP*	nucleoside antibiotic; acridine dye	27	0	Yes	0	0	0	0	0	0
3003576	*eptA*	peptide antibiotic	27	0	Yes	0	0	0	0	0	0

3003578	*pmrF*	peptide antibiotic	27	0	Yes	0	0	0	0	0	0
3000074	*emrB*	fluoroquinolone antibiotic	26	0	Yes	0	0	0	0	0	0
3000499	*acrE*	fluoroquinolone antibiotic; cephalosporin; cephamycin; penam	26	0	Yes	0	0	0	0	0	0
3000518	*crp*	macrolide antibiotic; fluoroquinolone antibiotic; penam	26	0	Yes	0	0	0	0	0	0
3000656	*acrS*	fluoroquinolone antibiotic; cephalosporin; glycylcycline; cephamycin; penam; tetracycline antibiotic; rifamycin antibiotic; phenicol antibiotic; triclosan	26	0	Yes	0	0	0	0	0	0
3002635	*aph(2")-IIa*	aminoglycoside antibiotic	26	0	Yes	0	0	0	0	0	0
3003548	*mdtN*	nucleoside antibiotic; acridine dye	26	0	Yes	0	0	0	0	0	0
3000254	*emrY*	tetracycline antibiotic	25	0	Yes	0	0	0	0	0	0
3001329	*mdtG*	fosfomycin	25	0	Yes	0	0	0	0	0	0
3002986	*bacA*	peptide antibiotic	25	0	Yes	0	0	0	0	0	0
3000216	*acrB*	fluoroquinolone antibiotic; cephalosporin; glycylcycline; penam; tetracycline antibiotic; rifamycin antibiotic; phenicol antibiotic; triclosan	24	0	Yes	0	0	0	0	0	0
3002688	*catS*	phenicol antibiotic	24	0	Yes	0	0	0	0	0	0
3000195	*tetB(P)*	tetracycline antibiotic	23	0	Yes	0	0	0	0	0	0
3000300	*lsaA*	lincosamide antibiotic; streptogramin antibiotic; pleuromutilin antibiotic	22	0	Yes	0	0	0	0	0	0

3000676	*H-NS*	macrolide antibiotic; fluoroquinolone antibiotic; cephalosporin; cephamycin; penam; tetracycline antibiotic	22	0	Yes	0	0	0	0	0	0
3003070	*vanXD*	glycopeptide antibiotic	22	0	Yes	0	0	0	0	0	0
3000180	*tetA(P)*	tetracycline antibiotic	21	0	Yes	0	0	0	0	0	0
3000593	*ermQ*	macrolide antibiotic; lincosamide antibiotic; streptogramin antibiotic	21	0	Yes	0	0	0	0	0	0
3002626	ant(6)-Ia	aminoglycoside antibiotic	21	0	Yes	0	0	0	0	0	0
3003069	*vanXYG*	glycopeptide antibiotic	21	0	Yes	0	0	0	0	0	0
3003206	*lsaE*	lincosamide antibiotic; pleuromutilin antibiotic	21	0	Yes	0	0	0	0	0	0
3000206	*emrK*	tetracycline antibiotic	20	0	Yes	0	0	0	0	0	0
3003551	*emeA*	acridine dye	20	0	Yes	0	0	0	0	0	0
3002923	*vanRD*	glycopeptide antibiotic	19	0	Yes	0	0	0	0	0	0
3002944	*vanHD*	glycopeptide antibiotic	19	0	Yes	0	0	0	0	0	0
3000005	*vanD*	glycopeptide antibiotic	18	0	Yes	0	0	0	0	0	0
3000522	*ermG*	macrolide antibiotic; lincosamide antibiotic; streptogramin antibiotic	17	0	Yes	0	0	0	0	0	0
3000263	*marA*	fluoroquinolone antibiotic; monobactam; carbapenem; cephalosporin; glycylcycline; cephamycin; penam; tetracycline antibiotic; rifamycin antibiotic; phenicol antibiotic; triclosan; penem	14	0	Yes	0	0	0	0	0	0
3000832	*evgA*	macrolide antibiotic; fluoroquinolone antibiotic; penam; tetracycline antibiotic	14	0	Yes	0	0	0	0	0	0
3002972	*vanTG*	glycopeptide antibiotic	14	0	Yes	0	0	0	0	0	0

3002630	*ant(9)-Ia*	aminoglycoside antibiotic	12	0	Yes	0	0	0	0	0	0
3000508	*gadX*	macrolide antibiotic; fluoroquinolone antibiotic; penam	11	0	Yes	0	0	0	0	0	0
3002882	*lmrD*	lincosamide antibiotic	10	0	Yes	0	0	0	0	0	0
3002957	*vanYD*	glycopeptide antibiotic	10	0	Yes	0	0	0	0	0	0
3001214	*mdtM*	fluoroquinolone antibiotic; lincosamide antibiotic; nucleoside antibiotic; acridine dye; phenicol antibiotic	7	0	Yes	0	0	0	0	0	0
3002909	*vanG*	glycopeptide antibiotic	6	0	Yes	0	0	0	0	0	0
3003112	*lsaC*	lincosamide antibiotic; streptogramin antibiotic; pleuromutilin antibiotic	6	0	Yes	0	0	0	0	0	0
3002881	*lmrC*	lincosamide antibiotic	4	0	Yes	0	0	0	0	0	0
3000792	*mdtA*	aminocoumarin antibiotic	3	0	Yes	0	0	0	0	0	0
3000186	*tetM*	tetracycline antibiotic	2	0	Yes	0	0	0	0	0	0
3000801	*mexD*	macrolide antibiotic; fluoroquinolone antibiotic; aminoglycoside antibiotic; cephalosporin; penam; tetracycline antibiotic; aminocoumarin antibiotic; diaminopyrimidine antibiotic; phenicol antibiotic	2	0	Yes	0	0	0	0	0	0
3003052	*smeB*	aminoglycoside antibiotic; cephalosporin; cephamycin; penam	2	0	Yes	0	0	0	0	0	0
3002629	*ant(6)-Ib*	aminoglycoside antibiotic	1	0	Yes	0	0	0	0	0	0

References

1. Meyer M, Kircher M. 2010. Illumina Sequencing Library Preparation for Highly Multiplexed Target Capture and Sequencing. Cold Spring Harb Protoc 2010:pdb.prot5448-pdb.prot5448.
